# A Minimal Regulatory Network of Extrinsic and Intrinsic Factors Recovers Observed Patterns of CD4+ T Cell Differentiation and Plasticity

**DOI:** 10.1371/journal.pcbi.1004324

**Published:** 2015-06-19

**Authors:** Mariana Esther Martinez-Sanchez, Luis Mendoza, Carlos Villarreal, Elena R. Alvarez-Buylla

**Affiliations:** 1 Departamento de Ecología Funcional, Instituto de Ecología, Universidad Nacional Autónoma de México, Coyoacán, México Distrito Federal, México; 2 Centro de Ciencias de la Complejidad, Universidad Nacional Autónoma de México, Coyoacán, México Distrito Federal, México; 3 Departamento de Biología Molecular y Biotecnología, Instituto de Investigaciones Biomédicas, Universidad Nacional Autónoma de México, México Distrito Federal, México; 4 Departamento de Física Teórica, Instituto de Física, Universidad Nacional Autónoma de México, México Distrito Federal, México; Virginia Tech, UNITED STATES

## Abstract

CD4+ T cells orchestrate the adaptive immune response in vertebrates. While both experimental and modeling work has been conducted to understand the molecular genetic mechanisms involved in CD4+ T cell responses and fate attainment, the dynamic role of intrinsic (produced by CD4+ T lymphocytes) versus extrinsic (produced by other cells) components remains unclear, and the mechanistic and dynamic understanding of the plastic responses of these cells remains incomplete. In this work, we studied a regulatory network for the core transcription factors involved in CD4+ T cell-fate attainment. We first show that this core is not sufficient to recover common CD4+ T phenotypes. We thus postulate a minimal Boolean regulatory network model derived from a larger and more comprehensive network that is based on experimental data. The minimal network integrates transcriptional regulation, signaling pathways and the micro-environment. This network model recovers reported configurations of most of the characterized cell types (Th0, Th1, Th2, Th17, Tfh, Th9, iTreg, and Foxp3-independent T regulatory cells). This transcriptional-signaling regulatory network is robust and recovers mutant configurations that have been reported experimentally. Additionally, this model recovers many of the plasticity patterns documented for different T CD4+ cell types, as summarized in a cell-fate map. We tested the effects of various micro-environments and transient perturbations on such transitions among CD4+ T cell types. Interestingly, most cell-fate transitions were induced by transient activations, with the opposite behavior associated with transient inhibitions. Finally, we used a novel methodology was used to establish that T-bet, TGF-β and suppressors of cytokine signaling proteins are keys to recovering observed CD4+ T cell plastic responses. In conclusion, the observed CD4+ T cell-types and transition patterns emerge from the feedback between the intrinsic or intracellular regulatory core and the micro-environment. We discuss the broader use of this approach for other plastic systems and possible therapeutic interventions.

## Author Summary

CD4+ T cells orchestrate adaptive immune responses in vertebrates. These cells differentiate into several types depending on environmental signals and immunological challenges. Once these cells are committed to a particular fate, they can switch to different cell types, thus exhibiting plasticity that enables the immune system to dynamically adapt to novel challenges. We integrated available experimental data into a large network that was formally reduced to a minimal regulatory module with a sufficient set of components and interactions to recover most CD4+ T cell types and reported plasticity patterns in response to various micro-environments and transient perturbations. We formally demonstrate that transcriptional regulatory interactions are not sufficient to recover CD4+ T cell types and thus propose a minimal network that induces most observed phenotypes. This model is robust and was validated with mutant CD4+ T phenotypes. The model was also used to identify key components for cell differentiation and plasticity under varying immunogenic conditions. The model presented here may be a useful framework to study other plastic systems and guide therapeutic approaches to immune system modulation.

## Introduction

The immune system protects organisms against external agents that may cause various types of diseases. As the immune system mounts specialized responses to diverse pathogens, it relies on plastic responses to changing immunological challenges. At the same time, the immune system must maintain homeostasis and avoid auto-immune responses. Therefore, the immune system relies on resilience mechanisms that enable it to return to basal conditions once pathogens or immunogenic factors are no longer present [1–3].

CD4+ T cells, also known as T helper (Th) cells, are key in the response to infectious agents and in the plasticity of the immune system. Naive CD4+ T cells (Th0) are activated when they recognize an antigen in a secondary lymphoid organ. Depending on the cytokine milieu and other signals in their micro-environment, CD4+ T cells attain different cell fates [2,4–7]. Nonetheless, we still do not have a complete understanding of the dynamic mechanisms underlying CD4+ T cell differentiation and plasticity [5].

**Fig 1:**
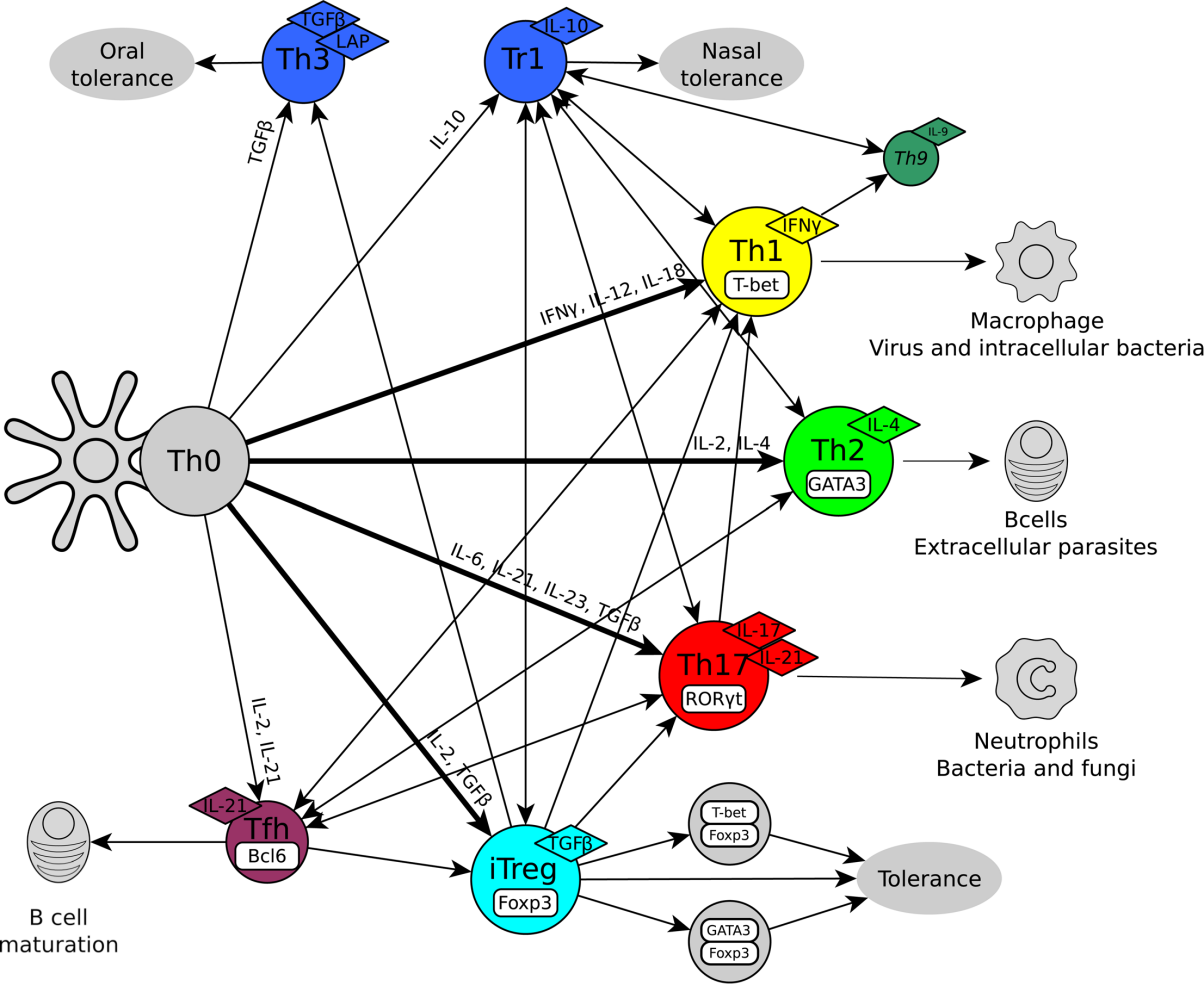
Differentiation and plasticity of CD4+ T cell types. CD4+ T cell types are characterized by their unique cytokine production profiles, transcription factors and biological functions. The main cell types are Th0, Th1, Th2, Th17, iTreg and Tfh. Other possible cell types have been described such as IL-9 (Th9), IL-10+Foxp3-(Tr1) and TGF-β+Foxp3-(Th3) producing cells.

Each CD4+ T cell type is associated with specific cytokines, receptors, transcription factors and functions (Fig 1). Th1 cells express T-bet, secrete interferon-γ (IFN-γ) and are associated with cellular immunity [8]. Th2 cells express GATA3, secrete interleukin (IL)-4 and are associated with immunity to parasites [8]. Th17 cells express RORα and RORγt, secrete IL-17 and IL-21, and are associated with neutrophil activation [9–10]. Follicular helper CD4+ T cells (Tfh) express Bcl6 and CXCR5, secrete IL-21 and are associated with B cell maturation in germinal centers [11,12]. Th9 cells secrete IL-9 and exert anticancer activity [13,14]. Induced regulatory T cells express Foxp3, secrete TGF-β and/or IL-10, and are associated with immune tolerance [15,16]. There is also considerable overlap among the expression profiles of different CD4+ T cells. For example, IL-9 and IL-10 can be secreted by Th1, Th2, Th17, iTreg cells and a variety of other immune cells [17–19]. T regulatory cells can also express IL-17 [20].

CD4+ T cells are highly plastic, switching from one type to another in response to environmental challenges (Fig 1) [1,21–23]. Th17 cells can transform into Th1 cells [24–25], and iTregs differentiate into Th17 in the presence of IL-6 [26]. Th2 cells can become IL-9 producing cells but may not easily become Th1 cells [27]. iTreg and Tfh cells can independently develop into other CD4+ T cell types, and they can be derived from Th1, Th2 or Th17 cells [28–30]. The differentiation and plasticity of CD4+ T cells depends on the interactions among the cytokines produced by other immune cells, epithelial cells, adipocytes, or by the CD4+ T cells themselves; the transduction of those signals and the regulation of this signaling by suppressors of cytokine signalling (SOCS) proteins; the set of transcription factors expressed inside the cells; epigenetic regulation; certain metabolites; and also microRNAs [4,6,31–33]. Given the complexity of CD4+ T cell transitions and the difficulty of classifying a particular expression pattern as a subset or a lineage, we will refer to the different stable expression patterns of CD4+ T cells as “cell-types”.

A mechanistic, integrative and system-level understanding of CD4+ T cell differentiation and plasticity requires dynamic regulatory network models that consider the concerted action of many components. These models can be used to prove whether the known biological interactions are necessary and sufficient to recover attractors that correspond to experimentally observed configurations in different CD4+ T cell types. Additionally, such models may be used to address whether the considered components and interactions also restrict and explain the observed patterns of transition among cell types. Finally, this type of model can be used to test the role of different network components in cell differentiation and plasticity.

In such regulatory network models, the nodes correspond to the regulatory components of the network such as genes, proteins or signals, while the links correspond to the interactions among components. The state of each node is determined by the expression level of its regulators, and the logical functions describe the dynamic evolution of the node states. The attractors, the states to which such regulatory networks converge, can be interpreted as the profiles characterizing different cell types (see reviews in: [34–36]).

Previous studies have used regulatory network models to study CD4+ T cell differentiation and plasticity [37–40]. These models captured the dynamic and non-linear regulation of CD4+ T cells and recovered the attractors corresponding to the Th0, Th1, Th2, iTreg and Th17 cell types. They have also been useful for preliminary studies of CD4+ T cell plasticity in the presence of different cytokines in the micro-environment [38] and fir studies of the effect of a specific molecule (PPARγ) in the Th17/iTreg switch [40]. However, as new T CD4+ cell types such as Tfh, regulatory Foxp3-independent, Th9, and Th22 cells are described, it is necessary to develop an updated regulatory network that is able to recover the configurations that characterize such novel cell subsets. Additionally, to date no minimal model that incorporates the necessary and sufficient set of interactions to also recover the reported patterns of transitions among Th cells has been reported.

Here, we specifically address whether CD4+ cell types and their transition patterns emerge as a result of the feedback between a minimal regulatory core of intra-cellular transcription factors and cytokines produced by the CD4+ T cell together with cytokines produced by other cells present in the micro-environment. Our results confirm that a regulatory network model that only considers the interactions among the master transcription factors is not sufficient to recover configurations that characterize the different CD4+ T cell types. Therefore, we then integrated a minimal network of master transcriptional factors with cytokine signaling pathway, including the cytokines produced by the cell and those present in the micro-environment, to integrate a network with the necessary and sufficient set of components to recover documented CD4+ T cell differentiation and plasticity patterns. The observed configurations of CD4+ T cells (Th0, Th1, Th2, Th17, iTreg, Tfh, Th9 and Foxp3-independent T regulatory cells) emerge from the feedback and cooperative dynamics among the multiple levels of regulation considered in the minimal model. In addition, this system is able to recover the plastic transition patterns and stability behavior that have been described for the different cell types in response to transitory perturbations and different micro-environments. Interestingly, our model predicts that transitions from particular cell types to others are caused by transient activations, while transient inhibitions usually cause cells to remain in their original cell types. Additionally, we show that T-bet, TGF-β and SOCS proteins are keys to recovering observed CD4+ T cell plastic responses. Finally, we discuss the relevance of our models for a system-level understanding of mammalian immunological responses and eventual biomedical interventions.

## Results

### CD4+ T cell regulatory network

Boolean networks are capable of integrating qualitative interactions (molecular, physical, chemical, etc.) into a coherent picture and are useful ways to explore the minimal set of restrictions that are necessary and sufficient to produce emergent biological patterns and behaviors [41–43]. The regulatory interactions considered in the present model are grounded on experimental data. In the proposed regulatory network, the nodes represent the regulatory components of the network and the links the interactions among them (S1 Table and S1 Fig). Given the complexity of the network, we simplified the model by removing intermediate components along a network path (S1 File) following a method proposed in [44] and checked the consistency of the reduced network using GINsim [45].

The predicted cell phenotypes arising from the steady states of the network are consistent with the available experimental data [2,4–7]. The model assumes that all interactions are synchronous, that all cytokine receptors are present, and that the TCR and its cofactors are activated (being unable to model unactivated and anergic CD4+ T cells). The model ignores weak interactions, low levels of expression, and epigenetic regulation (S1 File).

### A core of master transcriptional regulators is not sufficient to explain CD4+ T cell differentiation

**Fig 2:**
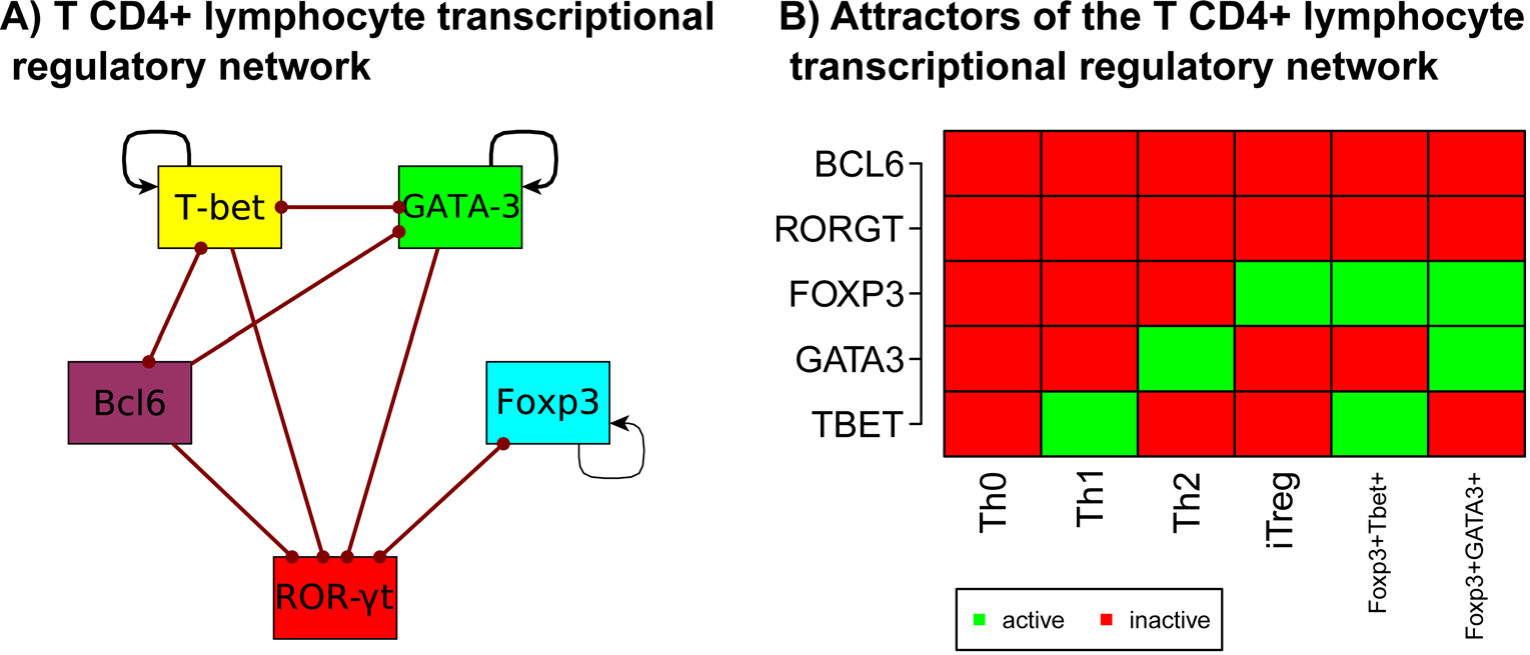
Minimal network of master transcriptional regulators CD4+ T (CD4+ T TRN). Based on published experimental data we constructed a CD4+ T cell regulatory network that includes the master transcriptional regulators and the interactions among those regulators (CD4+ T TRN). **(A)** Graph of the CD4+ T TRN. Node colors correspond to cell types: Th1 (yellow), Th2 (green), Th17 (red), iTreg (blue) and Tfh (purple). Activations among elements are represented with black arrows and inhibitions with red dotted arrows. **(B)** Attractors of the CD4+ T TRN: Each column corresponds to an attractor. Each node can be active (green) or inactive (red). The attractors correspond to configurations that characterize the Th0, Th1, Th2, iTreg, T-bet+Foxp3+ and GATA3+Foxp3+ types. The attractors corresponding to the Th17 and Tfh types could not be recovered.

To address whether a minimal transcriptional regulatory core could recover the observed configurations that characterize the main CD4+ T cell types that have been described up to now, we extracted from the general network under study a minimal regulatory module consisting only of transcriptional regulators (Fig 2A, S2 Table, BioModels Database: MODEL1411170000). Our aim was to test whether this minimal module contained a sufficient set of interactions to predict the observed configurations for the transcription factors included in the model that characterize different CD4+ T cell types. The nodes of the transcriptional regulatory network (TRN) correspond to the five “master” transcription factors associated with CD4+ T cell types: T-bet for Th1, GATA3 for Th2, RORγt for Th17, Foxp3 for iTreg, and Bcl6 for Tfh.

The dynamic analysis of this TRN recovered attractors corresponding to different CD4+ T cell types (Fig 2B): Th0, Th1, Th2, iTreg and the hybrid states T-bet+Foxp3+ [46] and GATA3+Foxp3+ [47]. However, this TRN did not converge to configurations that characterize the Th17 and Tfh cell types, implying that the expression of RORγt and Bcl6 is not sufficient to maintain such cell types. This result may be caused by the lack of feed-forward loops in the TRN. RORγt has no positive interactions with any of the transcription factors considered in the TRN and lacks a feedback loop mediated by transcription factors [48]. The mode of self-regulation of Bcl6 remains unclear, as it has been reported to either activate or inhibit its own expression in B cells [49–50].

### CD4+ T cell differentiation patterns emerge from feedback between the transcriptional regulatory network, cytokines and signaling pathways

The above result reveals which T CD4+ cell types rely only on the postulated TRN and which require extrinsic signals. To formally test this hypothesis, we extended the TRN network by introducing key components of signaling pathways and their regulators, as well as cytokines that have been shown to be fundamental in CD4+ T cell type attainment. This T CD4+ cell transcriptional-signaling regulatory network (TSRN) was then simplified (S1 File, S1 Fig) to obtain a minimal network. To reduce the number of nodes in the network, we assumed that the TCR signal was present and that the cytokine receptors were present in sufficient amounts to transduce a signal. This network lacks many important inflammatory cytokines (such as IL-1, TNFα), because while these cytokines are crucial for the immune response, they are dispensable for CD4+ T cell differentiation. The model analyzed in this paper also lacks extrinsic cytokines produced by other immune system cells and other cell types such as IL-12 and IL-18. The network also lacks some transcription factors and cytokines associated with newly reported Th types such as IL-22, as detailed experimental information linking them to the network model under analysis is not yet available.

**Fig 3:**
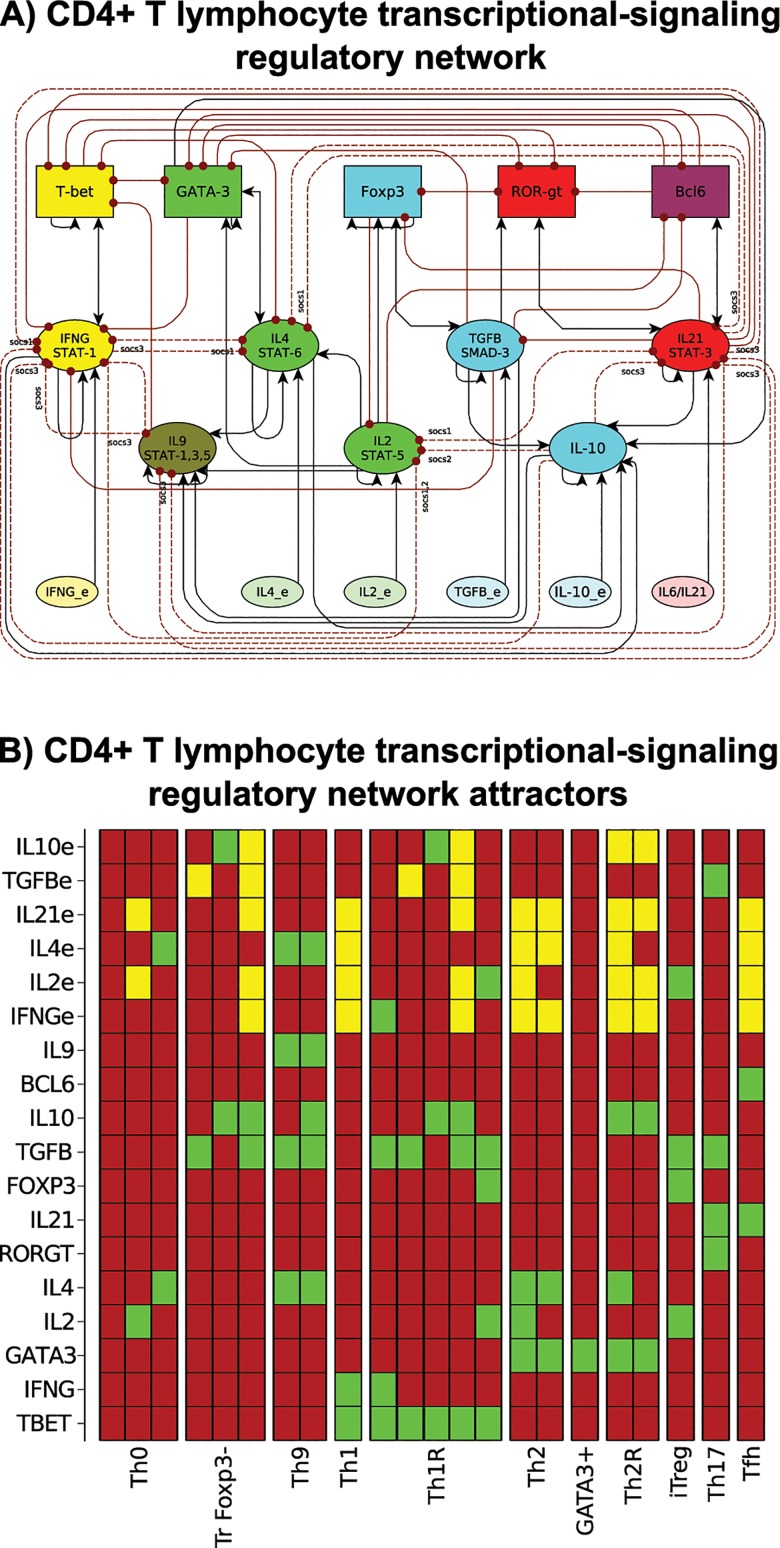
CD4+ T cell transcriptional-signaling regulatory network (TSRN). We constructed a regulatory network using available experimental data. The network includes transcription factors, signaling pathways, and intrinsic and extrinsic cytokines. **(A)** Graph of the TSRN. The nodes include transcription factors (rectangles), intrinsic cytokines and their signaling pathways (ellipses) and extrinsic cytokines (ellipses). Node colors correspond to cell type: Th1 (yellow), Th2 (green), Th17 (red), iTreg (blue), Tfh (purple), and Th9 (brown). Activations between elements are represented with black arrows, and inhibitions with red dotted arrows. The dotted lines represent inhibition mediated by SOCS proteins. **(B)** Attractors of the TSRN. Each column corresponds to an attractor. Each node can be active (green) or inactive (red), extrinsic cytokines may be active or inactive (yellow). The following attractors were found in the network: Th0, Th1, Th2, Th17, iTreg, Tfh, Th9 producing T cells, Foxp3-independent T regulatory cells (TrFoxp3-), T-bet+ T regulatory cells (Th1R), GATA3+ T regulatory cells (Th2R) and GATA3+IL-4- cells. Attractors where labeled according to the active transcription factors and intrinsic cytokines.

The nodes of the simplified TSRN represent (Fig 3A, S3 Table, BioModels Database: MODEL1411170001) transcription factors, signaling pathways and extrinsic cytokines. The nodes corresponding to cytokine pathways are active if the signal is transduced; this means that if the cytokine is present, it forms a complex with the receptor that can activate a messenger molecule (for example a STAT protein), which is then translocated to the nucleus. Cytokines can be produced by both CD4+ T cells (intrinsic) and by other cells of the immune system and the organism (extrinsic). To resolve this ambiguity we added nodes representing the extrinsic cytokines produced by other cells and tissues of the immune system (IL_e). This extended TSRN includes 18 nodes: the transcription factors (Tbet, GATA3, RORγt, Foxp3, Bcl6), the effector cytokines and their signaling pathways (IFN-γ, IL-2, IL-4, IL-21, IL-9), the regulatory cytokines (TGF-β and IL-10) and the extrinsic cytokines (IFN-γe, IL-2e, IL-4e, IL-21e, TGF-βe and IL-10e). While IL-10, IL-6 and IL-21 all signal using STAT3, IL-6 and IL-21 cause inflammation, while IL-10 suppresses inflammation. To analyze this network, we assume that IL-10 signaling was mediated by a different pathway than IL-6/IL-21, even though they share STAT3 as a messenger molecule. The production of these external cytokines is independent of regulation inside the CD4+ T cell, but their signaling can be blocked (for example by SOCS proteins [51]). The resulting network includes two levels of regulation, the regulation in the nucleus by mutually inhibiting transcription factors and the regulation among the receptors and their signal transduction pathways mediated by SOCS proteins.

The dynamic analysis of the TSRN yields stable configurations that correspond to: Th0, Th1, Th2, Th17, iTreg, Tfh, T regulatory Foxp3-independent cells, Th1R, Th2R and GATA3+IL4- cells (Fig 3B). As this biological patterns can be obtained in the presence of various extrinsic cytokines, we labeled each attractor according to the active transcription factors and intrinsic cytokines. Resting CD4+ T cells (labeled Th0) were defined as expressing no transcription factors or regulatory cytokines. Th1 was defined as Tbet and IFN-γ active [8], Th2 as GATA3 and IL-4 active [8] and GATA3+ (a Th2-like cell type) as GATA3+IL4-[38]. Th17 was defined based on RORγt and STAT3 signaling mediated by IL-6 or IL-21, all of which require the presence of TGF-βe [9–10]. iTreg expressed Foxp3 and TGF-β, IL-10 or both, all of which require the presence of IL-2e [16]. Interestingly, the TSRN model also predicts a novel set of steady states that had not been predicted by previous models but that correspond to reported biological cell types (Fig 3B); for example, Tfh cells with Bcl6 and STAT3 signaling mediated by IL-21 [12]; Th9 cells with IL-9, requiring the presence of TGF-β and extrinsic IL-4 [27]; T regulatory cells, as Foxp3-independent CD4+ T cells (TrFoxp3-) with TGF-β, IL-10 or both, but not Foxp3 [52]; Th1 regulatory cells (Th1R) expressing a regulatory cytokine and T-bet [46]; and Th2 regulatory cells (Th2rR) expressing a regulatory cytokine and GATA3 [47]. The model does not consider the Th22 cell type [53] because IL-22 was not included in the network due to the lack of experimental data on this molecule.

To validate the model with experimental data, we simulated loss and gain of function alterations for some nodes. In general, the results agree with the available experimental data, except in the case of the IL-2 knock-out. IL-2- causes the loss of iTreg cells as these cells require continuous IL-2 signaling [54,55], but this differs from the actual IL-2 KO mutants, which lose most CD4+ T cell types because IL-2 is also critical for the activation and survival of CD4+ T cells. This model also allows us to predict the behavior of the Tr Foxp3-, Th1R and Th2R cell types in response to various knock-out and over-expression simulations for several transcription factors or signaling pathways where no experimental data are available.

We performed a functional robustness analysis in which the logical functions of the network were altered (S2 Fig) to verify the construction of the functions and the structural properties of the model and to avoid over-fitting. Altering one of the functions of the network resulted in 1.389% of the initial states attaining a different final attractor than the original final state, and only 0.219% of the initial states arrived at an attractor that was not in the original set of attractors of the non-altered network.

To further verify that the results of the Boolean network are not an artifact due to the discrete nature of the model and to further assess the robustness of the attractors to variations in the node values, we approximated the discrete step-like functions of the Boolean model with continuous interaction functions [44] (S2 File). The continuous model recovers the same attractors as the Boolean regulatory network. Furthermore, these attractors are stable in response to small perturbations in the value of the nodes as predicted by the robustness analyses of the Boolean version of the model.

### CD4+ T cell differentiation in response to the micro-environment

**Fig 4:**
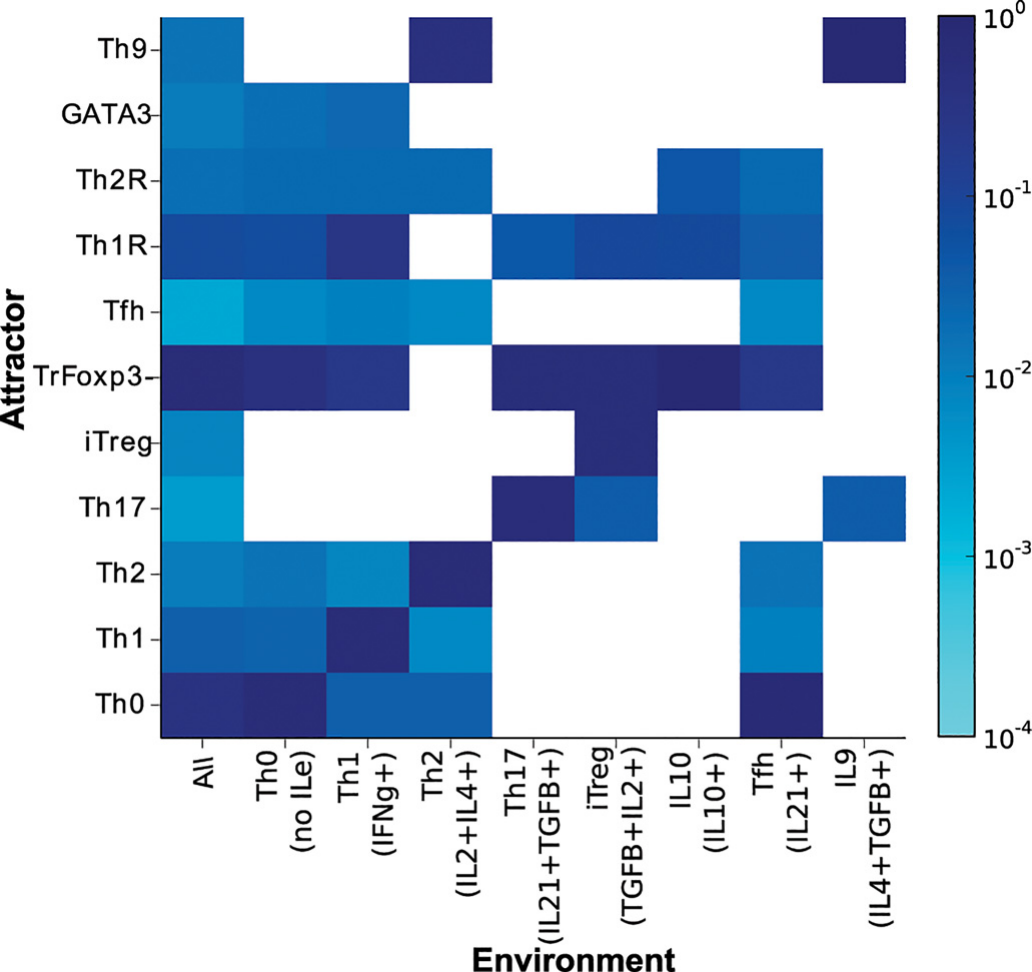
Effect of the micro-environment on CD4+ T cell differentiation as determined using the TSRN model. The values of the extrinsic signals of the TSRN were fixed according to different polarizing micro-environments. The basins of attraction of the resulting attractors were plotted on a logarithmic scale. The following micro-environments were studied: combinations of all extrinsic cytokines, no extrinsic cytokines (Th0), IFN-γe (Th1), IL-4e and IL-2e (Th2), IL-21e and TGF-βe (Th17), TGF-βe and IL-2e (iTreg), IL-10e (IL10), IL-21e (Tfh), and IL-4e and TGF-βe (Th9).

Cytokines can be produced by the cell (intrinsic) or by other cells of the immune system (extrinsic). These extrinsic cytokines constitute the micro-environment for CD4+ T cell differentiation. The role of polarizing micro-environments in CD4+ T cell differentiation was assessed using the TSRN model. In this network, the values of the extrinsic cytokines were fixed at a given expression level and the network response was analyzed again (Fig 4). Th0, Th1, Th2 and Tfh can be maintained in the absence of extrinsic cytokines or in the presence of effector cytokines such as IFN-γ, IL-2, IL-4 or IL-21. Th17, iTreg and Th9 cells require extrinsic TGF-β, IL-2 and IL-4, respectively, to maintain their homeostatic states [13,56]. TrFoxp3- states can be maintained in most polarizing micro-environments [57,58]. The recovered behaviors agree with the experimental data and also with previous models [38].

The importance of the extrinsic cytokines present in the micro-environment can be further analyzed when the system is studied under polarizing conditions. The presence of extrinsic signals for a given cell type increases the number of initial states that differentiate into that cell type, while the absence of extrinsic signals may lead to the loss of a cell type, as is the case with Th17, iTreg and Th9 cells (Fig 4). The presence of the regulatory cytokines IL-10 and TGF-β inhibits most effector CD4+ T cells, except for Th17. This finding may explain the presence of Th17 cells in regulatory micro-environments [59] and provides important insight concerning the relationship between Th17 and iTreg. Thus, this type of modeling framework and analysis may prove useful for finding therapeutic approaches to chronic inflammation.

The polarization of the micro-environment towards a particular cell type increases the size of the basin of attraction and its resistance to transient perturbations. Basin size and attractor stability are not identical (S3 Fig). In this way, the environmental signals promote specific cell types and increase their stability, which likely affects the population dynamics of CD4+ T cells. Nonetheless, different CD4+ T cell types coexist during immune responses. Even if the signals in the micro-environment promote a specific cell type, attractors corresponding to other cell types can still appear in this micro-environment, but their basin sizes and stability tend to be smaller.

### CD4+ T cell plasticity in response to the micro-environment

The ability of the immune system to dynamically respond to environmental challenges depends on its plastic responses. CD4+ T cells are phenotypically plastic, and once differentiated, their expression patterns can be altered depending on internal and external cues. This cell plasticity seems to be important for the overall plasticity of immune system responses [1].

**Fig 5:**
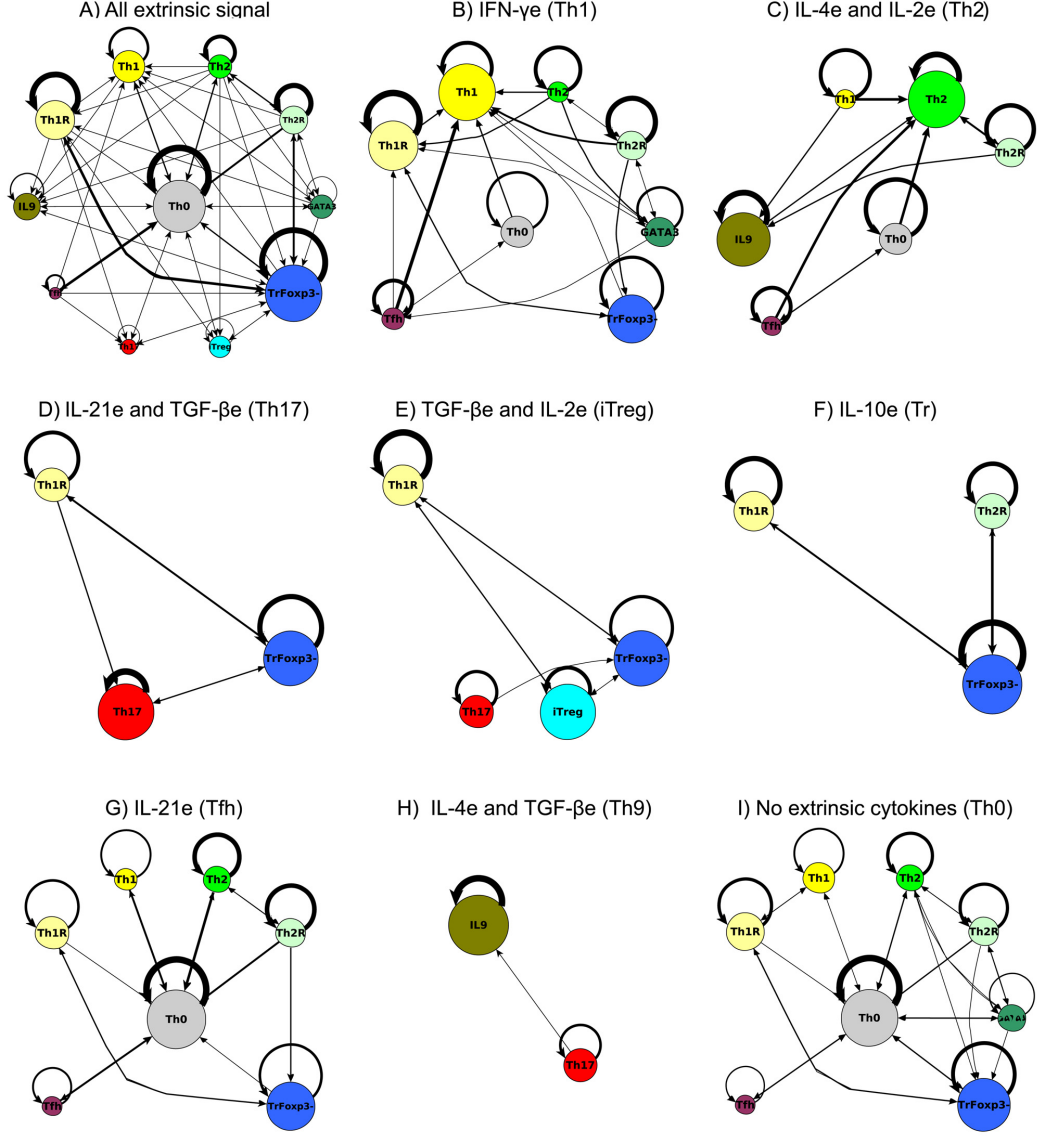
Cell fate map in response to the micro-environment and perturbations of the TSRN model. The values of the extrinsic signals of the TSRN were fixed according to different polarizing micro-environments, and the resulting attractors were transiently perturbed. The nodes represent CD4+ T cell types, and the node sizes correspond to the size of the basin of attraction. The edges represent transitions between cell types, the width of the edges corresponds to the number of times the transition occurred in logarithmic scale, and self-loops correspond to perturbations where the network returned to the original cell type. The following micro-environments were studied: combinations of: **(A)** all extrinsic cytokines, **(B)** IFN-γe (Th1), **(C)**IL-4e and IL-2e (Th2), **(D)** IL-21e and TGF-βe (Th17), **(E)** TGF-βe and IL-2e (iTreg), **(F)** IL-10e (IL10), **(G)** IL-21e (Tfh), **(H)** IL-4e and TGF-βe (Th9), **(I)** no extrinsic cytokines (Th0).

To analyze CD4+ T cell plasticity, we transiently perturbed the attractors of the system. For each attractor we altered the value of one of its nodes and then evaluated the system until an attractor was reached. If the original attractor was reached, we considered the corresponding cell type as stable towards that perturbation. If a new cell-type was reached, we considered that the transition from one cell type to another corresponded to phenotypic plasticity. This analysis was repeated for every node and every attractor. This methodology allowed us determine all the transitions between cell types, the specific perturbation that caused the transition, and the path from one cell-type to another. These transient perturbations in the values of the nodes are equivalent to developmental noise or temporal changes in the micro-environment of the cell. The result is a cell-fate map where the nodes represent CD4+ T cell types recovered by the TSRNand the connections represent the possible transitions between pairs of differentiated cell types (Fig 5, S3 File).

The model recovers the reported transitions corresponding to the polarization of naïve CD4+ T cells into canonical CD4+ T cell types, as well as various events of trans-differentiation between canonical CD4+ T cell types. Most of the predicted transitions are to or from Th0 or towards TrFoxp3-. It is important to clarify that the TCR complex was not included in the minimal model. Thus, in our model, the Th0 attractor represents resting CD4+ T cells. There are few direct transitions among the Th1, Th2, and Th17 cell types. The few direct transitions found towards iTreg and Tfh can only be achieved in polarizing micro-environments. It is also possible to transition from one of the main cell types to another one through the Th0, TrFoxp3-, Th1R, Th2R or GATA3+IL4- attractors. This ability raises multiple questions about the signals necessary for plasticity *in vivo*. It is possible that in order to transition from one cell type to another, some signals have to be maintained for a certain period of time, or that more than one perturbation is necessary. Further studies are required to determine which conditions are necessary and sufficient for CD4+ T cell type transitions to further understand CD4+ T cell plasticity.

Therefore, in the context of this study, we define plasticity as the potential of a given differentiated cell to attain other fates in response to alterations in the expression patterns of their intrinsic components and/or of the extrinsic micro-environment. Of the total of 121 possible transitions between cell types arising from those alterations, the TSRN network yielded 66 cell-type transitions. Thus, the topology or set of regulatory interactions proposed in this network generates restrictions in terms of cell types but also in terms of the patterns of cell-fate transitions.

CD4+ T cells are typically under the influence of particular micro-environments, with specific cytokines affecting the dynamics of these cells. Depending on the combination of cytokines, some cell types are lost, and transitions among the remaining cell types are also restricted. To simulate polarizing micro-environments, we fixed the value of the cytokines associated with pro-Th1 (IFGγe), pro-Th2 pro-(IL-4e, IL-2e), pro-Th17 (IL-21e, TGF-βe), pro-iTreg (TGF-βe, IL-2e), pro-Tr (IL-10,) pro-Tfh (IL-21e) and pro-Th9 (IL-4 and TGF-βe). In general, the polarizing micro-environment increases the size of the attraction basin, the stability and the transition into the attractor. The biological nature of the polarizing signal affects the nature of the resulting transition. In response to regulatory signals (IL-10e, TGF-βe), the majority of the transitions are towards TrFoxp3-, while inflammatory signals lead to more transition signals towards Th1 and Th2. All of these results represent interesting predictions that could be tested experimentally.

#### Activation of specific CD4+ T transcriptional-signaling regulatory network nodes induces cell type plasticity while inhibitions induce stability

**Fig 6:**
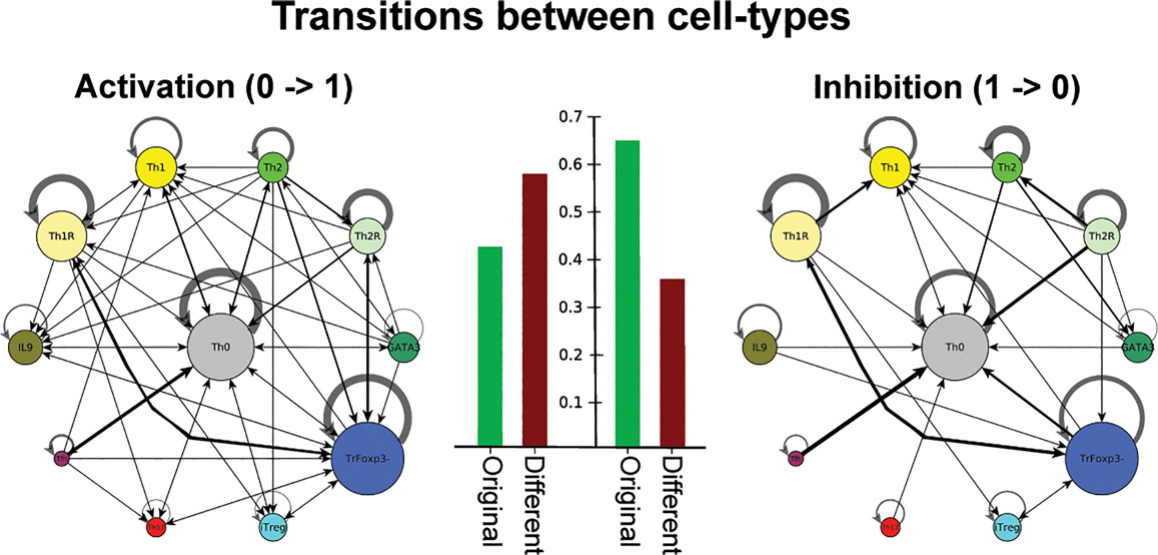
Cell fate map in response to activating or inhibitory signals of the TSRN model. The attractors of the network were transiently perturbed in all possible micro-environments. Perturbations were considered activations (0 → 1) when a previously inactive element was turned on, and inhibitions (1 → 0) when a previously active element was turned off. The nodes represent CD4+ T cell types, and the node sizes correspond to the size of the basin of attraction. The edges represent transitions between cell types, the width of the edges correspond to the number of times the transition occurred on the logarithmic scale. The number of transitions towards a different or the original cell type were counted for both activations and inactivations.

The nature of the perturbation is also important for CD4+ T cell plastic responses or stability (Fig 6). If an inactive node is activated (0→1), there is a high probability that a transition from one cell type to another is induced. In contrast, if an active node is inactivated (1→0), there is a high probability that the system remains in the original cell-type. This pattern may be caused by the topology of the network and, in particular, may depend on the functional feedback loops of the system that are altered. The positive feedback loops of a cell type may increase the stability of an attractor and help to recover a transiently inactive node, thus stabilizing a given differentiated state. We hypothesize that the activation of a previously inactive node may induce more transitions, as this alteration likely affects the positive and negative functional circuits of the system [60], thus increasing the chances that the system leads to a new attractor. Further simulations should be used to exhaustively test how specific regulatory circuits react to transient activations and inhibitions. In any case, the analysis presented in this study enables us to postulate that CD4+ T cells are expected to be able to react to activation signals and environmental alterations but are stabilized in response to the transient loss of signals. Thus, the proposed model for CD4+ T cell dynamics implies that these cells are under an unstable equilibrium between cell-fate stability and plasticity.

### Key nodes for CD4+ T transcriptional-signaling regulatory network plasticity

**Fig 7:**
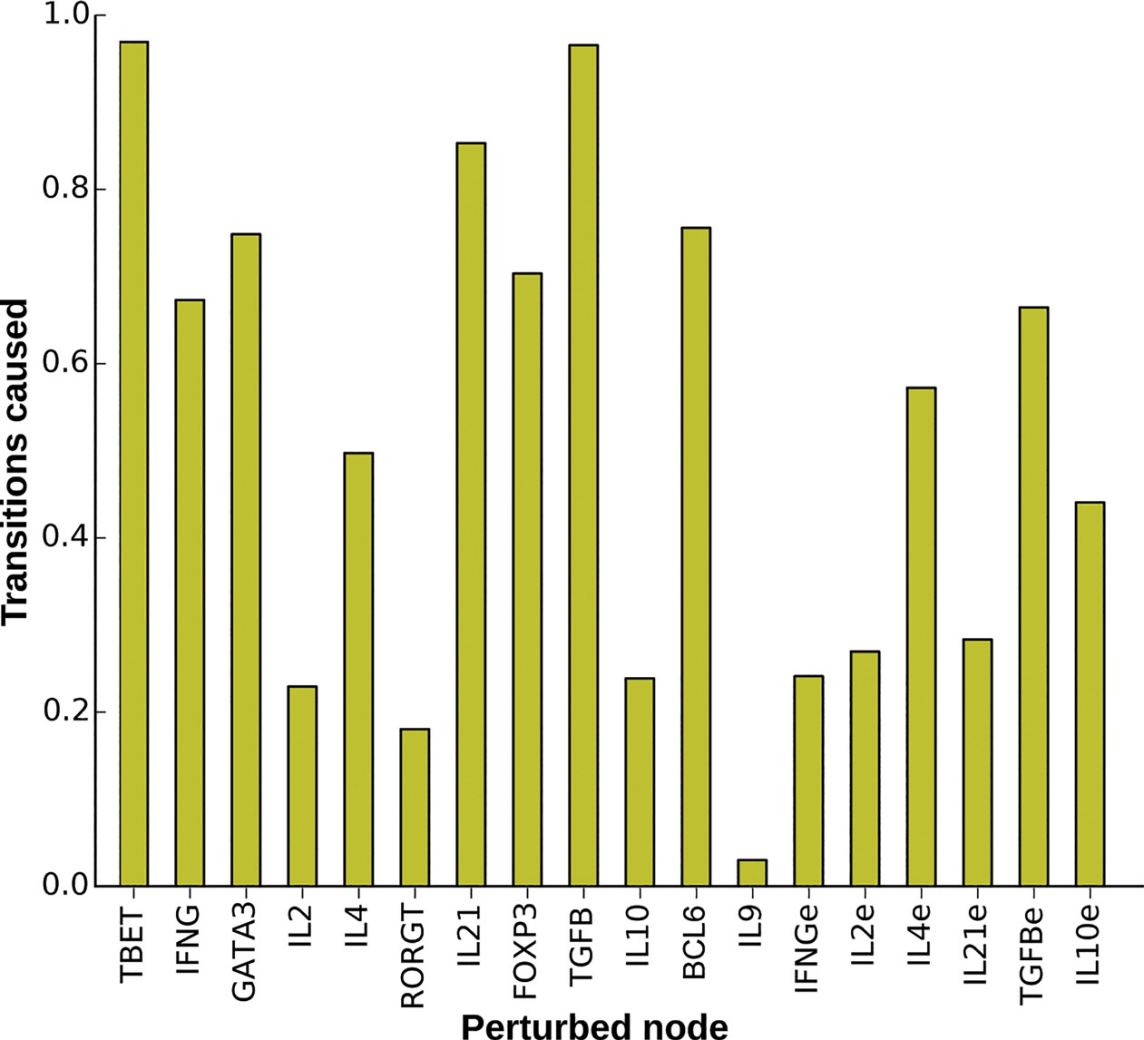
Role of different network nodes in the plasticity of the TSRN model. The proportion of transitions between attractors in response to transient perturbations in the value of each node. On average, 37.76% of the perturbations result in transitions to another cell type, with 47.12% of perturbations of intrinsic components resulting in transitions, compared with 24.43% of perturbations of extrinsic cytokines.

While all the elements of the TSRN have previously been shown to be necessary for the differentiation of CD4+ T cells, we wished to address their relative importance in cell plasticity responses. To evaluate this question, we perturbed each node of all the attractors and measured how many times the perturbed state changed to a new attractor (Fig 7) and to which new cell type the system converged (S4 Fig). This process is equivalent to the temporal activation or inactivation of a transcription factor or an element of the signaling pathway in response to noise. Alterations of T-bet and TGF-β usually caused the perturbed state to change from one attractor to another, while RORγt and IL-9 had the least effect on cell-fate transitions. In general, the system is more sensitive to perturbations in the master transcriptional regulators than to alterations of the cytokines.

**Fig 8:**
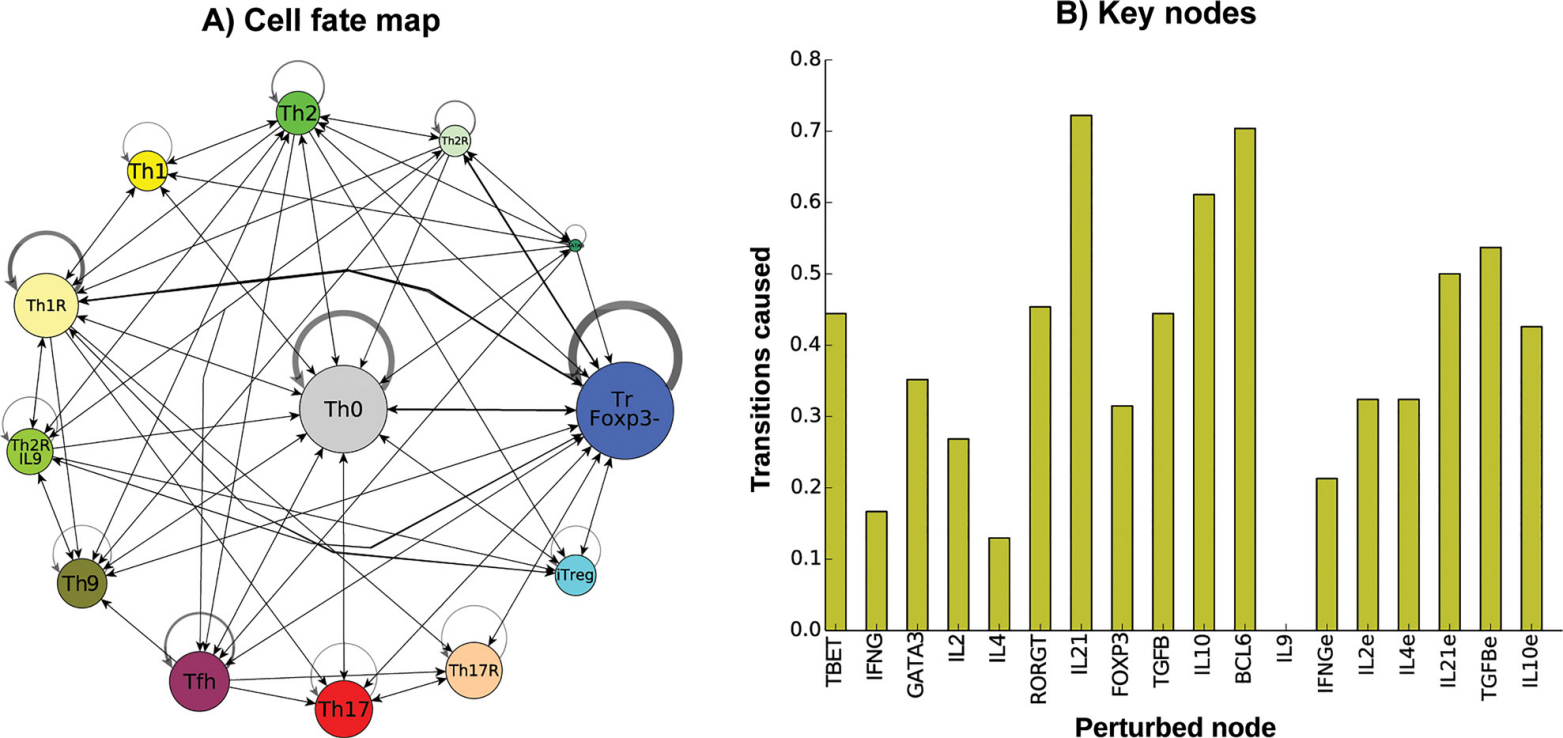
Role of SOCS proteins in the differentiation and plasticity of the TSRN model. The interactions mediated by SOCS proteins were removed to study their role. **(A)** Cell fate map of CD4+ T cell types when the SOCS protein interactions are removed from the TSRN model. The nodes represent CD4+ T cell types and the node sizes correspond to the size of the basin of attraction. New attractors corresponding to GATA3+IL9+IL10+ (Th2RTh9) and RORγt+IL-10+ (Th17R) appeared. The edges represent transitions between cell types, the width of the edges corresponds to the number of times the transition occurred on logarithmic scale, and self-loops correspond to perturbations where the network returned to the original cell type. **(B)** Proportion of transitions between cell types in response to transient perturbations in the value of each node. On average, 21.65% of the perturbations result in transitions to another cell type, with 17.55% of perturbations of the intrinsic components of the network resulting in transitions, compared with 27.51% of perturbations of extrinsic cytokines.

In contrast to previously published T CD4+ network models that only included SOCS1 [37–40], several SOCS-type proteins were considered in the TSRN presented and analyzed here. SOCS proteins are important for the differentiation and plasticity of CD4+ T cells. SOCS1 is commonly silenced in inflammatory diseases, and over-expression of SOCS3 correlates with allergies [31,51]. To explore the role of SOCS proteins and the impact of alterations in these proteins on CD4+ T type transitions, we generated a network lacking the inhibitions mediated by these proteins (Fig 8). This altered system recovers the original attractors including Th0, Th1, Th2, Th17, iTreg, Tfh, TrFoxp3-, and Th9, but it also predicts novel attractors expressing RORγt+IL-10+ (Th17R) and GATA3+IL-10+IL-9+ (Th2RIL9+), thus confirming the importance of SOCS proteins for attaining the Th17 and Th9 cell types. The importance of IL-10 for CD4+ T cell plasticity dramatically increased in the altered network, while the importance of the rest of the molecular elements decreased. This result suggests that SOCS proteins play an important role in stabilizing effector cell types and regulating the Th0 and TrFoxp3- cell types. SOCS proteins inhibit signal transduction; IL-10 in particular acts through these proteins to regulate CD4+ T cells. This regulation is important to buffer the effect of extrinsic cytokines in the TSRN network model. When SOCS proteins are absent, the network is more sensitive to changes in extrinsic cytokines and IL-10. Further analyses of the effects of SOCS proteins on CD4+ T cells and the possibility of updates to the model based on experimental work should enable the evaluation of more subtle alterations in and combinations of SOCS proteins.

## Discussion

This model provides a mechanistic description of the way in which CD4+ T cell types and plasticity emerge from the interactions among the intrinsic and extrinsic components of the immune response. The study formally shows that, as expected, the interactions among master transcription factors considered in the TSRN are not sufficient to recover the configurations characteristic of CD4+ T cell types, nor the reported transition patterns. Furthermore, these results clearly demonstrate the necessity to include the feedback from signaling pathways in response to cytokines to recover most of the range of CD4+ T cell types (Th0, Th1, Th2, Th17, Tfh, Th9, iTreg and T regulatory Foxp3 independent cells) and their transition pathways.

As noted above, CD4+ T cell differentiation does not arise solely from the regulatory action of the core of the reported "master" transcription factors (TF): T-bet, GATA3, Foxp3, RORγt and Bcl6. This may be due to the lack of feedforward loops mediated by the transcription factors RORγt [48] and Bcl6 [49,50]. These results show that the transcriptional regulatory core of CD4+ T cell differentiation is necessary, but not sufficient for CD4+ T cell differentiation. The emergence of the different CD4+ T cell types and their transition patterns, requires the feedback from cytokine signaling pathways and external cues.

This model provides a formal test for the emergence of different CD4+ T cell types from feedback or cooperative dynamics among master transcriptional factors, signaling pathway, cytokines produced by the cell and those present in the micro-environment. The proposed model recovers the observed configurations for the following CD4+ T cell types: Th0, Th1, Th2, Th17, Tfh, Th9, iTreg and T regulatory Foxp3 independent cells [2,4–7]. The model also yields the cell types Tfh, Th9 and T regulatory Foxp3 independent cells that had not been previously incorporated into such models [37–40].

CD4+ T cell types depend on signals from other cells for their differentiation and maintenance. The cytokines in the micro-environment restrict which cell types and transitions can be attained. A cytokine micro-environment that promotes a particular cell type increases its attraction basin size, stability and increases the number of transitions towards the promoted cell type. Nonetheless, different CD4+ T cell types can coexist in micro-environments that do not promote all the present cell types. For example, the presence of pro-regulatory cytokines IL-10 and TGF-β inhibits most effector cells, except for Th17. This finding may explain the presence of Th17 cells in regulatory micro-environments [59] and provides important insights concerning the relationship between Th17 and iTreg cells and the paradoxical role of TGF-β in inflammation [61]. Thus, the type of modeling framework and analyses presented here may prove to be useful for efforts to find therapeutic approaches to address chronic inflammation.

The model was also used to analyze the plasticity of CD4+ T cells by systematically testing how transient perturbations affect the transition patterns among cell types under various micro-environments. Previous studies focused on cell plasticity in response to different micro-environments [38] or on the role of specific molecules [40], rather than studying these phenomena as consequences of the global properties of the system. For example, the TSRN faithfully captures the polarization of resting CD4+ T cells into Th1, Th2, Th17, iTreg and Foxp3-independent T cells, but the predicted cell-fate maps lack direct transitions from iTreg to Th17 and Th17 to Th1 [23–25]. The TSRN model may lack components, interactions or epigenetic mechanisms of regulation that are important to enabling such direct transitions [33]. An additional possibility is that signals must be combined during particular lengths of time to enable some transitions. Further theoretical and experimental research is required to understand the mechanisms underlying CD4+ T cell plasticity. However, the qualitative model proposed here can serve as a framework to incorporate additional details involved in CD4+ T plasticity.

Our model shows that the activation of specific CD4+ T cell transcriptional-signaling regulatory network nodes generally induce cell type plasticity while inhibitions induce stability. The observed response patterns may be caused by the feedback loops and mutual inhibitions molecular network. These findings are coherent with the fact that the immune system generates a specific immunological response to particular challenges, maintains this response while the challenge remains present, and finally downregulates the immune response once the challenge has passed, thus maintaining homeostasis [3,61].

Our model suggests that T-bet, TGF-β and SOCS proteins are key network components to recover the observed CD4+ T cell plasticity. Although T-bet is a key transcription factor for Th1, it also inhibits other transcription factors regulating the differentiation into different cell types [4]. TGF-β is a critical regulator of the immune response but also plays a key role during chronic inflammatory responses [61]. SOCS proteins regulate the phosphorylation of STAT proteins, playing a key role in modulating the signal transduction among different cell types [31,51]. Determining the key elements enabling cell-type plasticity has possible therapeutic implications, as these findings can help to identify therapeutic targets for modulating the immune response while predicting and avoiding secondary effects[3,62].

Given the complexity of CD4+ T cell expression patterns and transitions, it remains unclear whether cytokine expression profiles correspond to lineages or subsets [1–3,22]. The term lineage implies the stability of the cellular phenotype and that the cell has committed to an expression pattern and will maintain it in a fairly robust manner, regardless of environmental alterations. On the other hand, the term subset implies that the cell has a specified expression pattern but that extrinsic signals are required to maintain that pattern [1,22]. Cell types Th1, Th2, Tfh and TrFoxp3- can be considered lineages, as they exhibit commitment under different cytokine milieus, even if the extrinsic signals change, although environmental alterations can still affect their stability. However, Th17, iTreg and Th9 cells, which require TGF-βe, IL-2e or IL-4e respectively, are potentially subsets. Th17 and iTreg cells also have small basins of attraction, low stability, and require extrinsic signals, exhibiting a lack of commitment. Th9 has a larger basin of attraction than Th17 or iTreg, but is less stable and susceptible to environmental alterations. Based on our analyses, we propose that the degree of dependence on extrinsic signals and the stability in response to changes in the micro-environment can provide clearer and more objective criteria to distinguish between CD4+ T cell subsets and lineages.

CD4+ T cell differentiation and plasticity arises from the feedback among multiple levels of regulation: transcriptional regulation, signaling pathways and the micro-environment. Studying the molecular network as a dynamic system allows us to understand how the interactions among the components, the topology of the network, and the dynamic functions of the nodes give rise to the biological behavior. However, further theoretical and experimental research is required to understand CD4+ T cells. As our understanding of these cells improves, it will be possible to incorporate more detailed molecular information, such as the effect of relative expression levels and the characteristic time courses of expression in the system. This will, in turn, allow us to recover novel cell types and their relationship with other CD4+ T cell types and other cells of the immune system. The present model can now be extended to incorporate multiple cells and their population dynamics [39], relationships with other cells of the immune system, and the formation of specialized niches that result from the dynamic interaction with the micro-environment. This approach will allow us to differentiate between CD4+ T cell subsets and lineages, to understand the developmental dynamics between the different cell types, and to propose approaches to immune system reprogramming that can be used in the clinic.

## Methods

### Logical modeling formalism: Boolean networks

CD4+ T cell differentiation results from interactions among cytokines, signaling pathways and transcription factors. These interactions were modeled using Boolean networks that enabled us to integrate the qualitative nature of complex regulatory systems. A Boolean network is composed of nodes that represent the system´s molecular components (i.e., cytokines, signaling pathways or transcription factors). In a Boolean network, each node represents a component (gene, protein, phenomenological signal) that can be associated with a discrete variable denoting its current functional level of activity. If the node is functional its value is 1, and if it is not functional, then its value is 0 (see S1 File). Some nodes required special considerations concerning their activation states in the Boolean model. For example, in the case of GATA3, which is continuously expressed during T-cell-lineage development and is necessary for lineage commitment and maintenance, GATA3^low^ is set to 0. As GATA3 is upregulated in Th2 differentiation [63], we set GATA3^high^ to 1. Another example concerns STAT proteins, which are activated when the protein is phosphorylated, forming a dimer that translocates to the nucleus, where it activates its target genes. In this case, the value for STAT protein activation was only set to 1 when all the required conditions were met.

The value of a node xi at a time t depends on the value of the input nodes (including itself), referred to as its regulators. This value can be expressed with a logical function that describes the behavior of the node through time:

$$x_{i}(t)=\phi_{\iota }(\tau ,\xi_{1},\xi_{2},\xi_{2},\ldots ,\xi_{\iota },\ldots ,\xi_{\nu }).$$

Weak interactions that are not necessary or sufficient, but only modulate a target factor, were not included in the input regulators of the truth tables (S1 File). Such is the case for Foxp3, which positively modulates the expression of IL-2Rα, which can be activated and functional in the absence of Foxp3 [64].

An input is a node that affects the values of the network but is independent of the network. The state of the network S can be represented by a vector that specifies the value of each node. The state of the network can be represented by a vector S composed of the values of all the nodes of the system. The state of the network corresponds to the expression patterns of a cell.

#### Inference of the regulatory functions

Boolean functions were defined based on the available experimental data for the reported interactions among a network of 85 components (S1 Table). A transcription factor regulates another factor if it binds to the regulatory region of the latter factor and inhibits or activates is transcription. A cytokine is present if it is either secreted by the cell (intrinsic) or produced by other cells of the immune system (extrinsic). To separate the effects of the cytokines produced by the immune system from those of the cytokines produced by the CD4+ T cell, we label extrinsic cytokines as ILe. Receptors are considered to be active when the cytokine is stably bound to a receptor, enabling it to transduce a signal. STAT proteins are considered active when they are phosphorylated and capable of translocating to the nucleus. The activation of a STAT protein depends on the presence of interleukin, its correct binding to the receptor, and subsequent phosphorylation. SOCS proteins inhibit the phosphorylation of STAT by competing for the phosphorylation site.

### Model reduction

To facilitate the analysis of the network and determine which components were necessary and sufficient to recover observed profiles and their patterns of transition, we reduced the extended regulatory network consisting of 85 nodes to one with 18 nodes, including 5 transcription factors, 7 signaling pathways and 6 extrinsic cytokines. To simplify the network, we assumed that the signal produced by the TCR and its co-factors was constitutive and ignored weak interactions as well as input and output nodes. Considering that the expression level of node x_i_ at time t is represented by x_i_(t), the attractors (steady states) that represent different phenotypes are determined by: x_i_(t+1) = x_i_(t).

In that case, the mapping becomes a set of coupled Boolean algebraic equations. The explicit expressions of the attractors are then obtained by performing the algebraic operations according to the axioms of Boolean algebra [44]. Self-regulated nodes were not removed. If a node was removed, then the logical rules of its targets were modified, maintaining the regulatory logic and indirect regulation. To verify that we did not remove a necessary node, we recovered the attractors of the network and ensured that the configurations corresponding to the Th0, Th1, Th2, Th17 and iTreg states could still be attained (see the details of the reduction methods used in S1 File).

The reduction was verified using the GINsim[45] software. GINsim uses decision diagrams to iteratively remove regulatory components and updates the components to maintain the indirect effects. This method preserves the dynamic properties of the original model. The simplification with GINsim returned a similar network to the one that we obtained with the Boolean logic reduction method proposed by Villareal et al. ([44];S1 File).

### Dynamic analysis

After inferring and simplifying the network, we studied its dynamic behavior. A regulatory network is a dynamic system. The state of a network will change over time depending on the logical functions associated with each node. When the values of a state vector S at t+1 are the same as those at time t, the system has attained an attractor: S*(*t*) = *S*(*t* + *n*), *n* ≥ 1.

An attractor is interpreted as a stable expression phenotype of a cell, representing a cell type. All the states that lead to a solution S* constitute the basin of attraction of such an attractor. We determined the attractors and basins of attraction of the network using the R library BoolNet. Attractors were classified depending on the expression of both the master transcription factors and the main cytokine. Th0 was defined as expressing no transcription factors or regulatory cytokines. Th1 was defined as Tbet and IFN-γ active [8], Th2 as GATA3 and IL-4 active [8] and GATA3+ (a Th2-like cell type) as GATA3+IL4-[38]. Th17 was identified by RORγt and STAT3 signaling mediated by IL-6 or IL-21, all of which require the presence of TGF-βe [9–10]. The iTreg type was defined by Foxp3 and TGF-β, IL-10 or both, all of which require the presence of IL-2e [16]. Tfh cells were defined by Bcl6 and STAT3 signaling mediated by IL-21 [12]. Th9 cells express IL-9, requiring the presence of TGF-β and extrinsic IL-4 [27]. T regulatory Foxp3-independent CD4+ T cells (TrFoxp3-) featured TGF-β, IL-10 or both, without expressing Foxp3 [52]. Th1 regulatory cells (Th1R) express a regulatory cytokine and T-bet [46]. Th2 regulatory cells (Th2rR) express a regulatory cytokine and GATA3 [47].

#### Network validation

The network was validated by comparing it with reported knock-out and over-expression profiles. To simulate loss of function mutations (knock-out) and inhibitions of the signaling pathway, we set the value of the corresponding node to 0 throughout the complete simulation. To simulate over-expression, the value of the node was set to 1.

The functional robustness of the network was characterized by altering the logical functions of the network. Functional robustness refers to the invariance of the attractors in response to noise or perturbations [44]. In this case, to verify that the results of the model did not depend on over-fitting the logical functions, we perturbed the latter and verified the stability of the resulting attractors and their basins. To achieve this, we randomly selected a large number of entries and flipped their values from 0 to 1 or vice versa, one by one (bit flip). The basins and attractors were obtained for the altered networks and compared the original basins and attractors.

To further evaluate the robustness of the network to small changes in the values of the nodes and interaction functions, we approximated the Boolean step functions as continuous functions [44]. We replaced the logical functions f(x_i_) with a set of continuous functions that satisfy Zadeh's rules of fuzzy propositional calculus. Using this approach for each state variable, we derived a continuous function, w_i_(q). The latter functions correspond to step-like (differentiable) activation functions. The continuous system can then be described by:

$$\frac{dq_{i}}{dt}=\frac{1}{e^{[-2b(w_{i}(q)-w_{i}^{thr})]+1}}18$$

where w_i_ is the input function for node i, w_i_^thr^ is a threshold level, b is the input saturation rate, and α_i_ is its relaxation rate. In particular, for b >>1, the activation function becomes a Heaviside step function.

### Plasticity

The attractors of the network correspond to cell types. A multi-stable system can have multiple attractors and switch between them in response to alterations in the state of the system [65]. To study the plasticity and robustness of the system we transiently perturbed the attractors of the network and then evaluated the functions until we arrived at an attractor. This methodology enabled us to obtain all the transitions between cell types, the specific perturbations that caused those transitions, and the path from one cell-type to another. We define an attractor as stable when the system remains in the same attractor in the presence of perturbations. The stability of each attractor in response to changes in the micro-environment and signaling pathways was analyzed by characterizing the evolution of the network in response to pulses of activation or inhibition of specific nodes. To quantify the stability of the attractors of the network, we perturbed the state vector of the solutions for one time step. Then, we counted how many of the perturbed state vectors stayed in the same attractor to quantify its stability. A system is plastic when it can transition from one state to another in response to alterations of the system. More specifically, the network was said to be plastic when a transition occurred from a given attractor to another in response to a transient perturbation in the value of one of its nodes.

## Supporting Information

S1 Table T CD4+ lymphocyte extended regulatory network references. (XLS)

S2 Table T CD4+ lymphocyte transcriptional regulatory network model. (XLS)

S3 Table T CD4+ lymphocyte transcriptional-signaling regulatory network model. (XLS)

S1 File T CD4+ lymphocyte extended regulatory network simplification. (PDF)

S2 File T CD4+ lymphocyte transcriptional-signaling continuous regulatory network model. (PDF)

S3 File Transitions in response to transient perturbations in the nodes of the T CD4+ lymphocyte transcriptional-signaling regulatory network. (PDF)

S1 Fig T CD4+ lymphocyte extended regulatory network. (EPS)

S2 Fig Validation of the T CD4+ lymphocyte transcriptional-signaling regulatory network. (A) To validate the TSRN model, we simulated loss of function or null mutations (KO) and over-expression experiments and compared the results with the available experimental data. The values of the nodes were set to “0” for simulations of loss-of-function or knock-out experiments and to “1” for over-expression. The color corresponds to the basin size of each attractor on the logarithmic scale. '—-' represents attractors that were not attained in the original wild type (WT) network. The attractors marked with (red) "X" correspond to incorrect predictions. (B) To verify the construction of the functions and the structural properties of the model, we performed a robustness analysis altering the update rules. Networks with perturbed functions of the TSRN were generated to test the robustness of the structural properties of the networks to noise, mis-measurements and incorrect interpretations of the data. After altering one of the functions of the network, 1.389% of the possible initial states changed their final attractor (yellow), and only 0.219% of the possible initial states arrived at an attractor not present in the original network (red).
(EPS)

S3 Fig Effect of the environment on the stability of the T CD4+ lymphocyte transcriptional-signaling regulatory network. The values of the extrinsic signals of the TSRN were fixed according to different polarizing micro-environments. Each attractor was transiently perturbed, and the proportion of transitions that stayed in the same cell type was plotted on a logarithmic scale. The following micro-environments were studied here: combinations of all extrinsic cytokines, no extrinsic cytokines (Th0), IFN-γe (Th1), IL-4e and IL-2e (Th2), IL-21e and TGF-βe (Th17), TGF-βe and IL-2e (iTreg), IL-10e (IL10), IL-21e (Tfh), and IL-4e and TGF-βe (Th9).
(EPS)

S4 Fig Effect of transient perturbations on the state of the nodes of the T CD4+ lymphocyte transcriptional-signaling regulatory network. Number of transitions to an attractor in response to transient perturbations in the value of each node. The states of the node were perturbed during one time step from 1 to 0 (-) or 0 to 1 (+), depending on its state in the original attractor.
(EPS)
