# Supplementary material for: A Minimal Regulatory Network of Extrinsic and Intrinsic Factors Recovers Observed Patterns of CD4+ T Cell Differentiation and Plasticity
Source: PLoS Comput Biol. 2015 Jun 19;11(6):e1004324. doi: 10.1371/journal.pcbi.1004324 (PMC4475012; doi:10.1371/journal.pcbi.1004324)
Supplement: S1 File — (PDF) [file pcbi.1004324.s004.pdf]

Supplementary Material
Network Simplification

Mariana Martinez-Sanchez Luis Mendoza Carlos Villarreal
Elena R. Alvarez-Buylla

The differentiation and plasticity of CD4+ T lymphocytes is the result of the concerted action of many components like cytokines, receptors, transcription factors, etc. The large number of components makes it convenient to simplify the resulting network.

1 Construction of the logical functions

We considered that a node is active if there is enough amount of protein or gene expression to be functional and affect the differentiation of CD4+ T lymphocytes. A transcription factor is active if it is present in enough quantity and in a conformation that can alter the expression of its target genes. A transcription factor or cytokine is active if it is present in enough quantity and in a conformation that can form a functional complex with its receptor. A receptor is active if it forms a complex that can activate its downstream signaling. A STAT proteins is active if it is phosphorylated and forms a dimer capable of translocating to the nucleus and affecting the expression of its target genes.

Basal levels

A protein or gene may be expressed at a basal level, but does not necessarily affect the differentiation of the cell at that level of expression. For example, GATA3 is necessary for T cell maduration and for CD4+ T-cell survival and maintenance. The deleterious mutation of GATA3 is letal, and Lck-Cre conditional deletion models lack CD4+ T cells or have impaired survival and maintainance. GATA3^{high} also drives the differentation into Th2 (Ho, Tai and Pai 2009). In this case we considered that the basal level of GATA3^{low} corresponded to zero, while GATA3^{high} was one.

Expression	Phenotype	Node value
GATA3^{KO}	Letal	-
GATA3^{low}	Survival	0
GATA3^{high}	Th2	1

Weak interactions

Weak interactions were ignored in our model. Interactions between genes and proteins are weak when they increase or decrease the expression of a gene or protein but are not necessary or sufficient to cause changes in differentiation. For example, The IL-2 receptor (IL2-R) is necessary for the activation of CD4+ T cells and plays a central tole in the differentiation towards Th2 and iTreg. IL-2R is composed of three subunits IL-2Rα, IL-2Rβ, γ_c. The three subunits together form a high affinity receptor, while IL-2Rβ and γ_c form a medium affinity receptor, both complexes are

functional. IL2 increases the expression of IL-2Rα and IL-2Rβ and Foxp3 increases the expression of IL-2Rα. The result is that the IL-2R can form a functional complex (IL2R = 1) in the presence of IL-2 with or without Foxp3, even if the transcription factor affects its expression levels and affinity (Liao 2011).

IL-2_t	Foxp3_t	IL2-R_{t+1}
0	0	0
0	1	0
1	0	1
1	1	1

2 Boolean Logic Reduction Method

To simplify the network we employed a Boolean reduction method proposed in Villarreal *et al*, 2012. For simplicity, we illustrate only the simplification scheme of the interactions between IL-2 and Foxp3. Interleukin 2 (IL-2) can be produced by the T CD4+ lymphocytes or by other cells of the immune system (IL2e). IL-2 binds the IL-2 receptor (IL-2R), which causes the phosphorylation and dimerization of STAT5. The phosphorylation of STAT5 can be inhibited by SOCS1, which binds the IL-2R. STAT5 activates the transcription of IL-2, Foxp3 and increases the transcription of IL-2R. Foxp3 can induce its own transcription and inhibit the transcription of IL-2. These interactions can be characterized by a set of logical propositions which satisfy the following mapping:

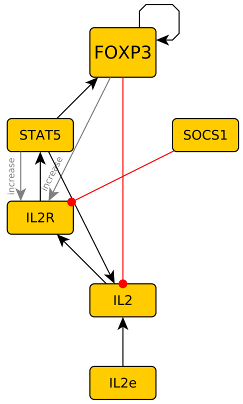

$$\begin{aligned} IL2_{t+1} &= IL2e_t \text{ or } (STAT5_t \text{ and not } FOXP3_t) \\ IL2R_{t+1} &= IL2_t \text{ and not } SOCS1_t \\ STAT5_{t+1} &= IL2R_t \\ FOXP3_{t+1} &= STAT5_t \text{ and } FOXP3_t \end{aligned} \tag{1}$$

Considering that the expression level of node N at a time t is represented by N_t the attractors (steady states) that represent different phenotypes are determined by the condition $N_{t+1} = N_t$. In that case, the mapping becomes a set of coupled Boolean algebraic equations. The explicit expressions of the attractors are then obtained by performing the algebraic operations according to the axiomatic of Boolean algebra (see Villarreal *et al*, 2012):

$$\begin{aligned} IL2 &= IL2e \text{ or } (STAT5 \text{ and not } FOXP3) \\ IL2R &= IL2 \text{ and not } SOCS1 \\ STAT5 &= IL2R \\ FOXP3 &= STAT5 \text{ and } FOXP3 \end{aligned} \tag{2}$$

This results in the identity:

$$STAT5 = IL2R \tag{3}$$

We employ this identity to determine the system's attractors:

$$STAT5 = IL2 \text{ and not } SOCS1 \tag{4}$$

$$STAT5 = (IL2e \text{ or } (STAT5 \text{ and not } FOXP3)) \text{ and not } SOCS1 \tag{5}$$

Thus, the regulatory network attractors are summarized by the expression values of the nodes pertaining to a concise set of Boolean expressions:

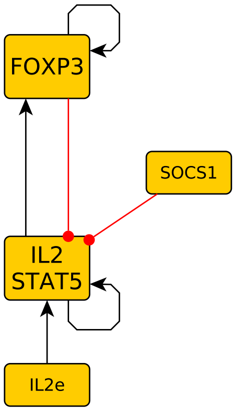

$$\begin{aligned} STAT5 &= (IL2e \text{ or } (STAT5 \text{ and not } FOXP3)) \text{ and not } SOCS1 \\ FOXP3 &= STAT5 \text{ and } FOXP3 \end{aligned} \tag{6}$$

3 Reduction of logical regulatory graphs

To verify the Boolean approach we compared our results with those obtained with the software GINsim (Naldi *et al*, 2009). GINsim uses decision diagrams to iteratively remove regulatory components and actualizes the components to maintain the indirect effects. The method preserves the dynamical properties of the original model. The simplification with GINsim returns a similar network as the one obtained with the boolean logic reduction method.

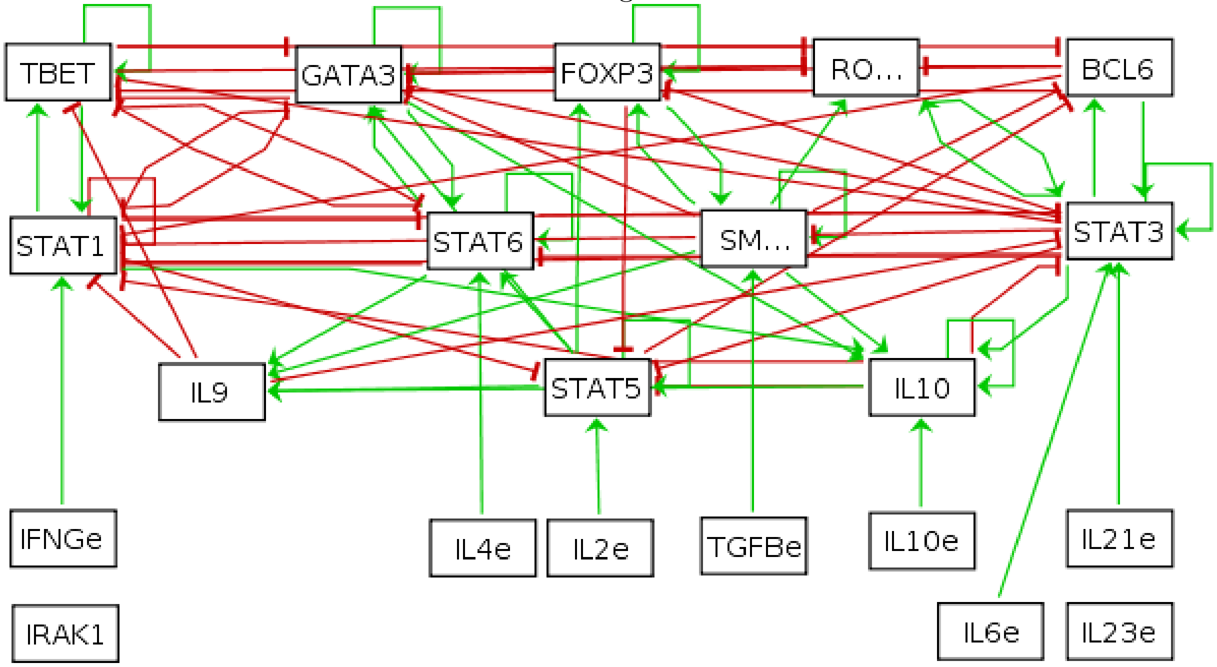

The simplification with GINsim recovers the same attractors as the Boolean logic reduction method, except for RORγt+STAT1+, Bcl6+STAT1+, and Foxp3+IL4+IL9+ attractors. The RORγt+STAT1+ and Bcl6+STAT1+ attractors arise from the codification of inhibitory interactions; our model assumes that inhibitions are strong, while GINsim assumes that enough activators may overcome an inhibition. The Foxp3+IL4+IL9+ attractor requires both IL2e and IL4e; under transient perturbations it transits towards Th9 (IL10+, IL2e-, IL10e+), iTreg (IL4e+), Th1R (IFNGe-) or Tfh (Bcl6+, IL21e+).

Name	TBET	GATA3	RORGT	FOXP3	BCL6	STAT1	STAT5	STAT6	STAT3	SMAD3	IL10	IL9	IRAK1	IFNGe	IL2e	IL4e	IL6e	IL21e	IL23e	TGFBe	IL10e
													*						*		*
										1			*						*	*	
										1	1		*		*			1	*	*	*
										1	1		*		*		1	*	*	*	*
								1					*			1	*		*		*
								1		1		1	*			1	*		*	*	
								1		1	1	1	*		*	1	*	*	*	*	*
							1						*		*				*		*
					1				1				*		*	*	*	*	*		
					1	1			1				*	1	*	*	*	*	*		
				1			1			1			*		1				*	*	
				1			1	1		1		1	*		1	1	*		*	*	
			1						1	1			*		*	*	*	*	*	1	
			1			1			1	1			*	1	*	*	*	*	*	1	
		1											*						*		
		1									1		*		*			1	*		*
		1									1		*		*		1	*	*		*
		1						1					*			*	*		*		
		1						1			1		*		*	*	*	*	*		*
		1					1	1					*		*	*	*		*		
	1									1			*						*	*	
	1									1	1		*		*			1	*	*	*
	1									1	1		*		*		1	*	*	*	*
	1					1							*	1	*	*	*		*		
	1					1					1		*	1	*	*	*		*		*
	1					1				1			*	1	*	*	*		*	*	
	1					1				1	1		*	1	*	*	*		*	*	*
	1			1			1			1			*		1				*	*	
