# Supplementary material for: A Minimal Regulatory Network of Extrinsic and Intrinsic Factors Recovers Observed Patterns of CD4+ T Cell Differentiation and Plasticity
Source: PLoS Comput Biol. 2015 Jun 19;11(6):e1004324. doi: 10.1371/journal.pcbi.1004324 (PMC4475012; doi:10.1371/journal.pcbi.1004324)
Supplement: S2 File — (PDF) [file pcbi.1004324.s005.pdf]

# Supplementary Material
# Network Simplification

Mariana Martinez-Sanchez  Luis Mendoza  Carlos Villarreal
Elena R. Alvarez-Buylla

Boolean GRNs are useful to study the complex logic of transcriptional regulation involved in cell differentiation. However, a comprehensive understanding of the mechanisms participating in cell fate dynamics must take into account the continuos character of the variables involved in the description: levels of genetic expression, differences in concentrations, in decay rates, threshold expression values, etc. These factors may be taken into account by translating the discrete dynamical mappings describing GRN interactions into a set of differential equations. In order to get formal consistency of both descriptions, the new variables and operators that constitute the logical propositions must satisfy a generalization of Boolean axiomatics into the continuous realm. For that purpose, we transform the logical connectors *and*, *or*, and *not* according to the following operations:

$$a \textit{ and } b \to a \cdot b \qquad a \textit{ or } b \to a + b - a \cdot b \qquad \textit{not } b \to 1 - b. \tag{1}$$

It is straightforward to show that these rules satisfy the axioms of Boolean algebra. We may then transform the Boolean logical propositions by direct substitution. An example is given by:

$$(a \textit{ or } b) \textit{ and not } c \to [a + b - a \cdot b] \; (1 - c).$$

We now consider the following set of differential equations defined by step-like inputs $\Theta[w_i]$, where $w_i$ is a continuous logical proposition:

$$\frac{dx_i}{dt} = \Theta\left[w_i(x_1, ..., x_n) - \theta_i\right] - \alpha_i x_i. \tag{2}$$

Here, $\theta_i$ is a threshold value (usually, $\theta_i = 1/2$), while $\Theta\left[w_i(x_i - \theta_i)\right]$ is a logistic functional whose value is 1 if $w_i > \theta_i$, 1/2 if $w_i = \theta_i$, and 0 if $w_i < \theta_i$. $\alpha_i$ represents the decay rate for the expression of node $i$. A representation of $\Theta[w_i]$ is

$$\Theta[w_i] = \frac{1}{\exp\left[-\beta(w_i - \theta_i)\right] + 1}, \tag{3}$$

where $\beta$ is a saturation rate. For $\beta \gg 1$, the functional $\Theta[w_i]$ becomes a Heaviside step function: $\Theta[w_i - \theta_i] \to H[w_i - \theta_i]$.

It may be shown that when all $\alpha_i = 1$, the steady states of the set (2), defined by $dx_i/dt = 0$, coincide with attractor set provided by the discrete Boolean approach, indicating the robustness of the continuous analysis.

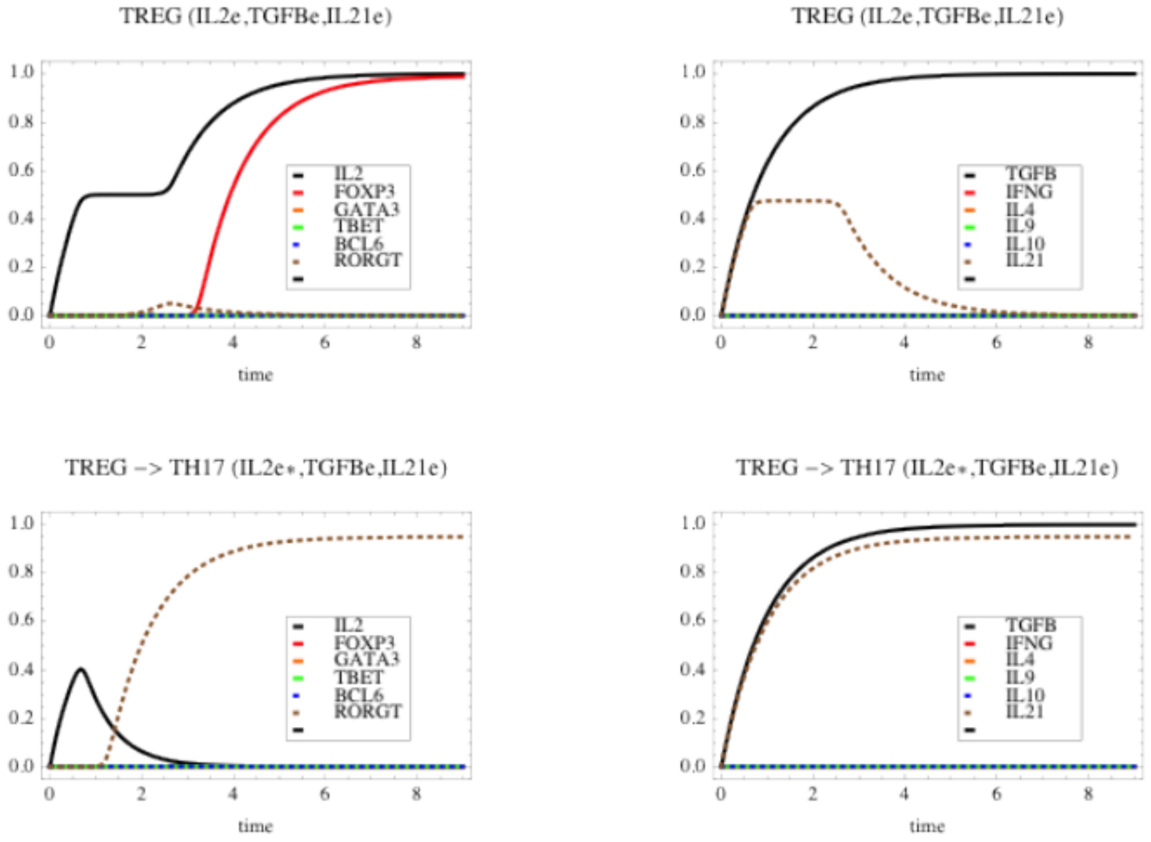


Figure 1: Differentiation and plasticity of CD4+ T cells in the continuous model. (A & B) Differentitation of Treg cells in response to IL2e, TGFBe and IL21e in the micro-environment. (C & D) Transition from Treg to Th17 in response to perturbations in IL2e.
