# Supplementary material for: A Minimal Regulatory Network of Extrinsic and Intrinsic Factors Recovers Observed Patterns of CD4+ T Cell Differentiation and Plasticity
Source: PLoS Comput Biol. 2015 Jun 19;11(6):e1004324. doi: 10.1371/journal.pcbi.1004324 (PMC4475012; doi:10.1371/journal.pcbi.1004324)
Supplement: S3 File — (PDF) [file pcbi.1004324.s006.pdf]

Transitions in response to transient perturbations in the nodes of the T CD4+ minimal regulatory network

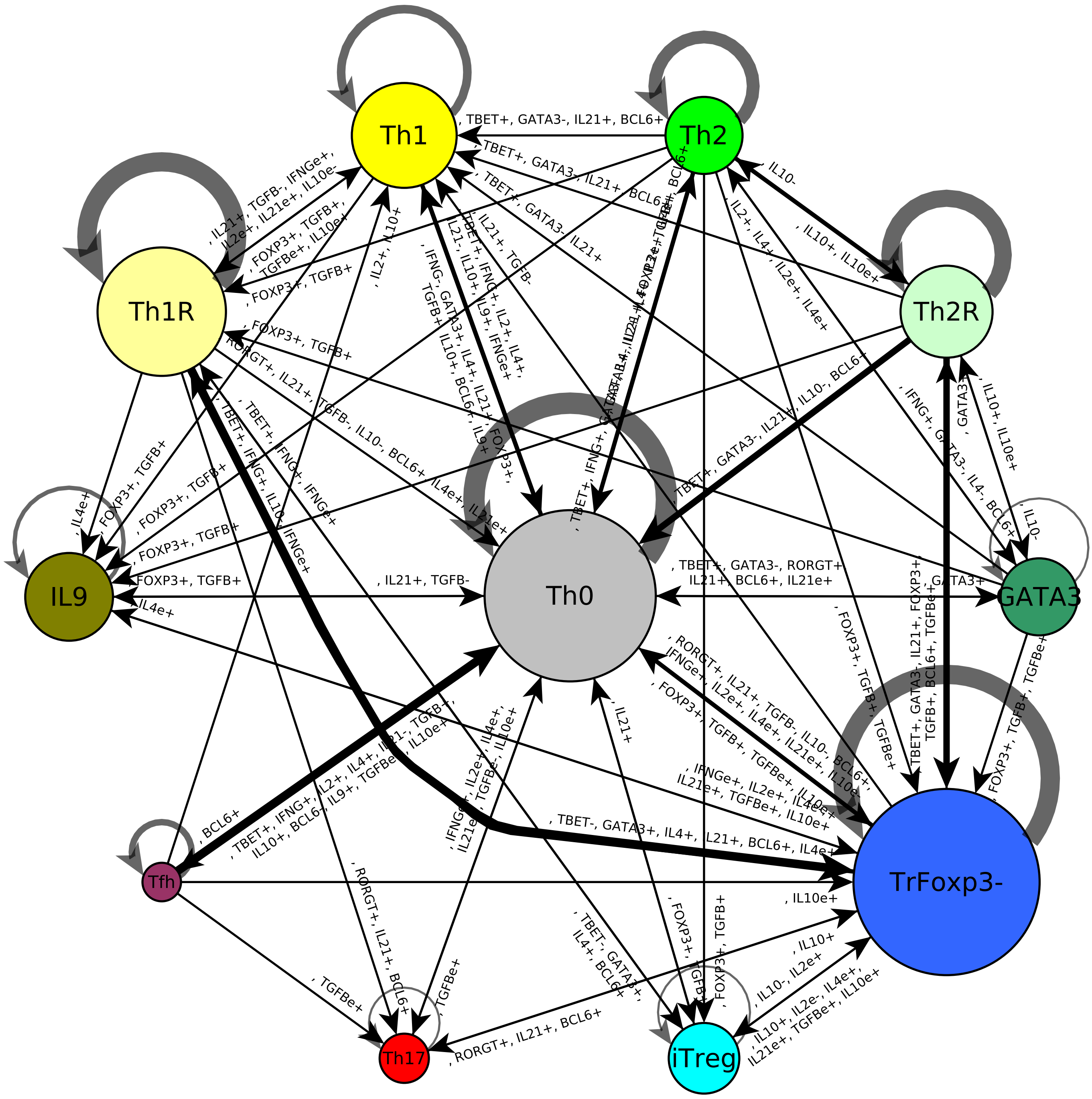

Th0 (No extrinsic signals)

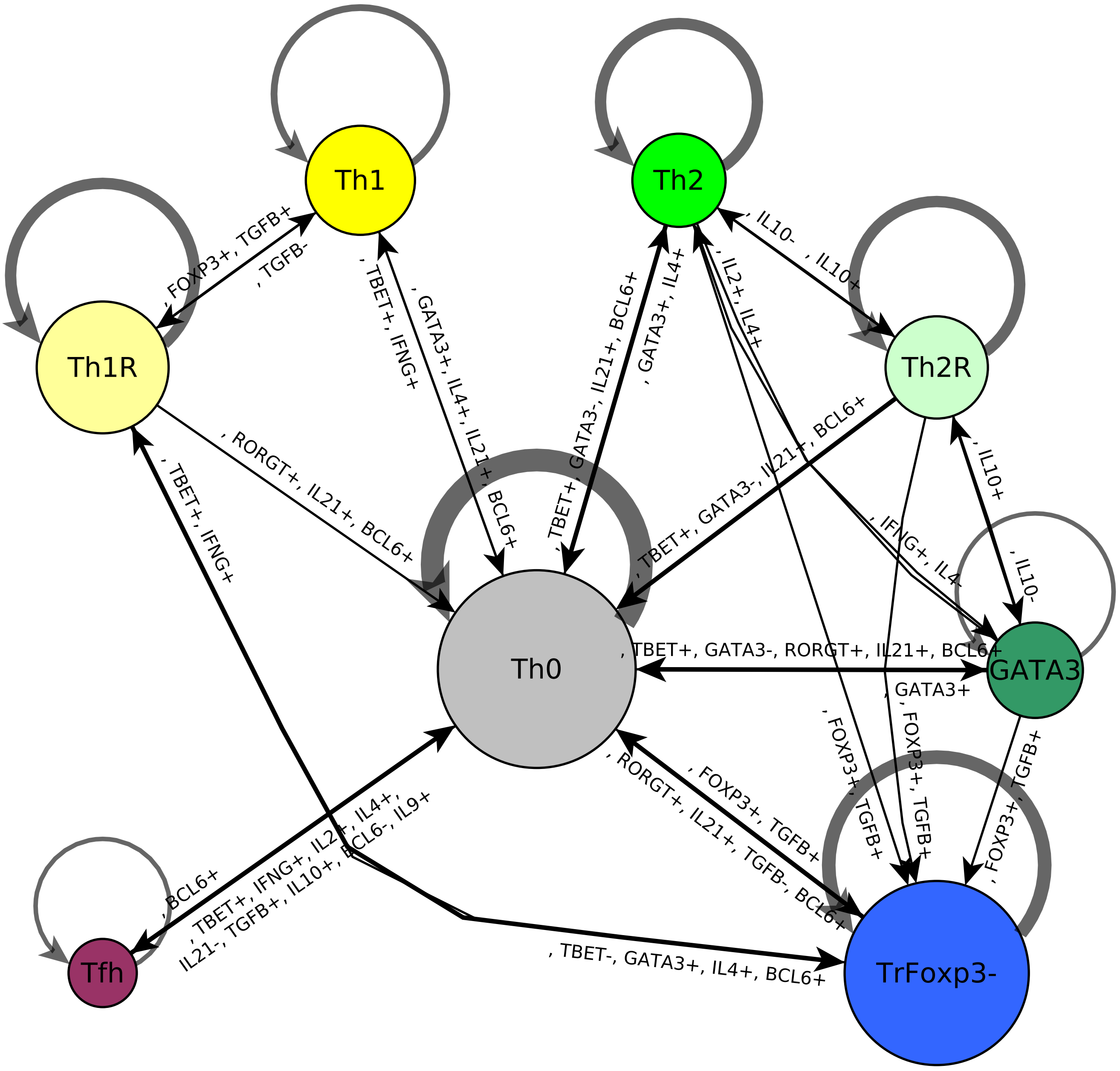

Th1 (IFNγe+)

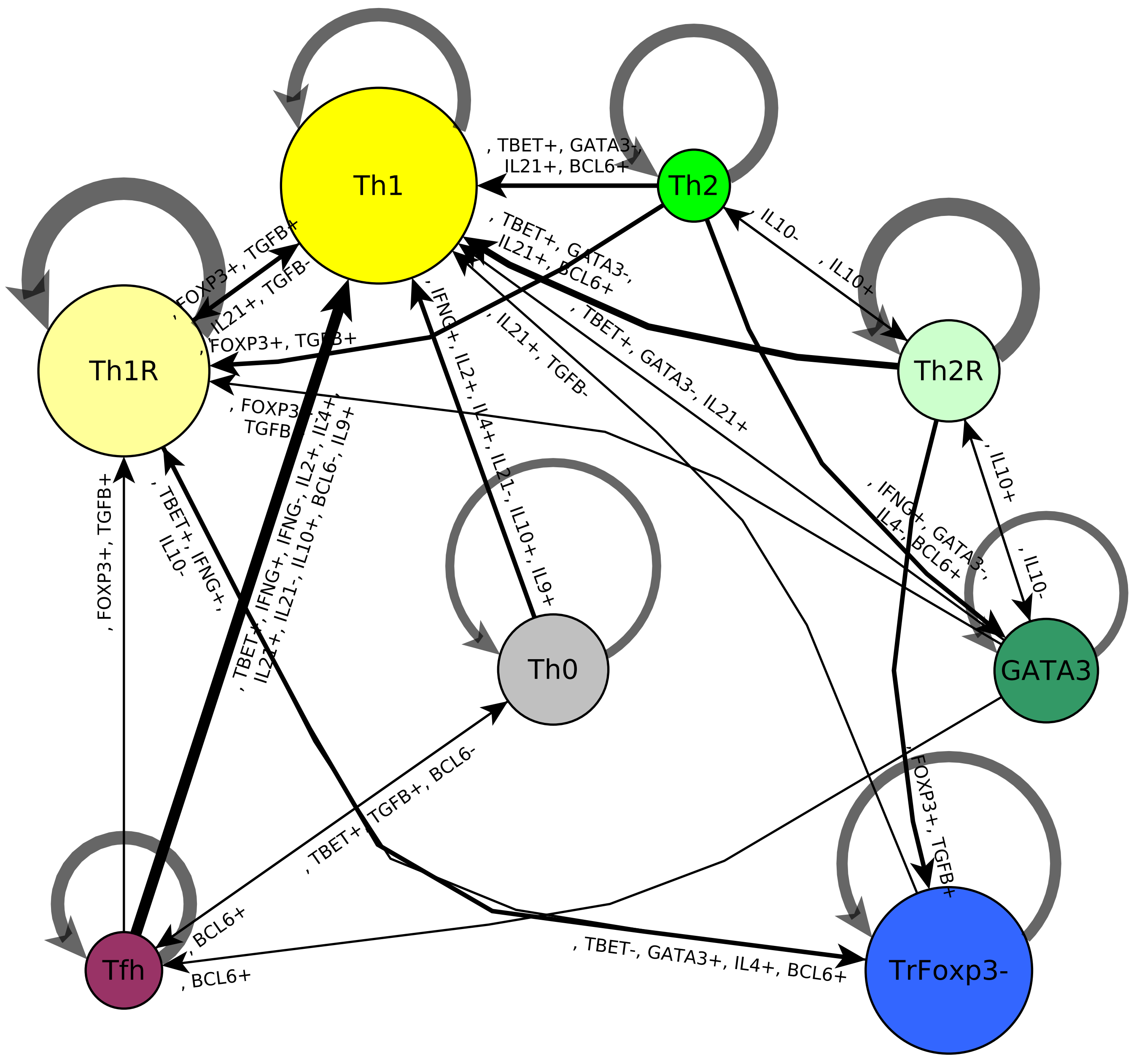
Th1
Th2
Th1R
Th2R
Th0
GATA3
Tfh
TrFoxp3-
, TBET+, GATA3-, IL21+, BCL6+
, TBET+, GATA3-, IL21+, BCL6+
, IL10-
, IL10+
, FOXP3+, TGFB+
, IL21+, TGFB-
, FOXP3+, TGFB+
, IFNG+, IL2+, IL4+, IL21-, IL10+, IL9+
, IL21+, TGFB-
, TBET+, GATA3-, IL21+
, IFNG+, GATA3-, IL4-, BCL6+
, IL10+
, IL10-
, FOXP3+, TGFB+
, TBET+, IFNG+, IFNG-, IL2+, IL4+, IL21+, IL21-, IL10+, BCL6-, IL9+
, TBET+, IFNG+, IL10-
, FOXP3+, TGFB+
, TBET+, TGFB+, BCL6-
, BCL6+
, BCL6+
, TBET-, GATA3+, IL4+, BCL6+
, FOXP3+, TGFB+

Th2 (IL-4e+ and IL-2e+)

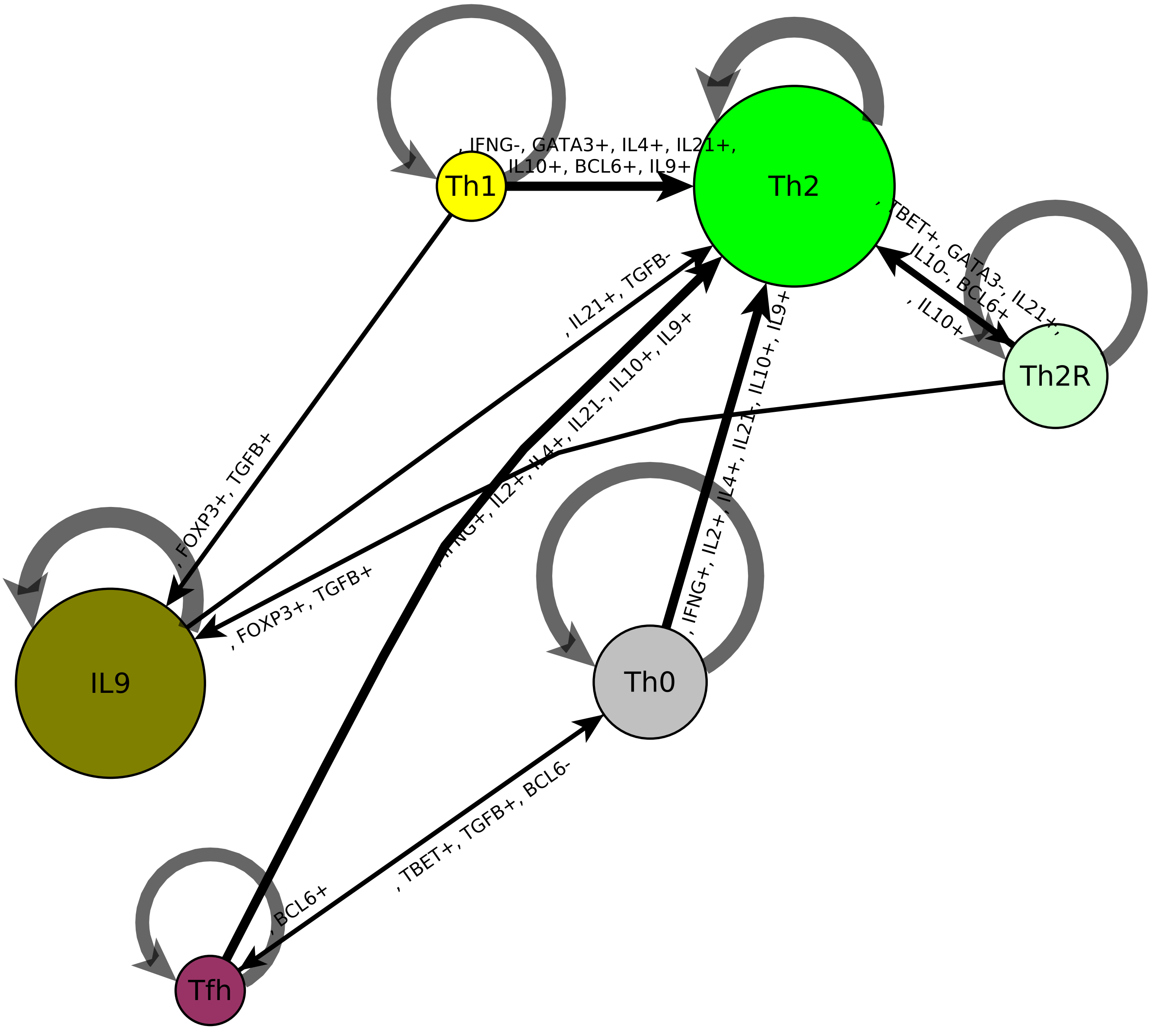

Th17 (IL-21e+ and TGFβe+)

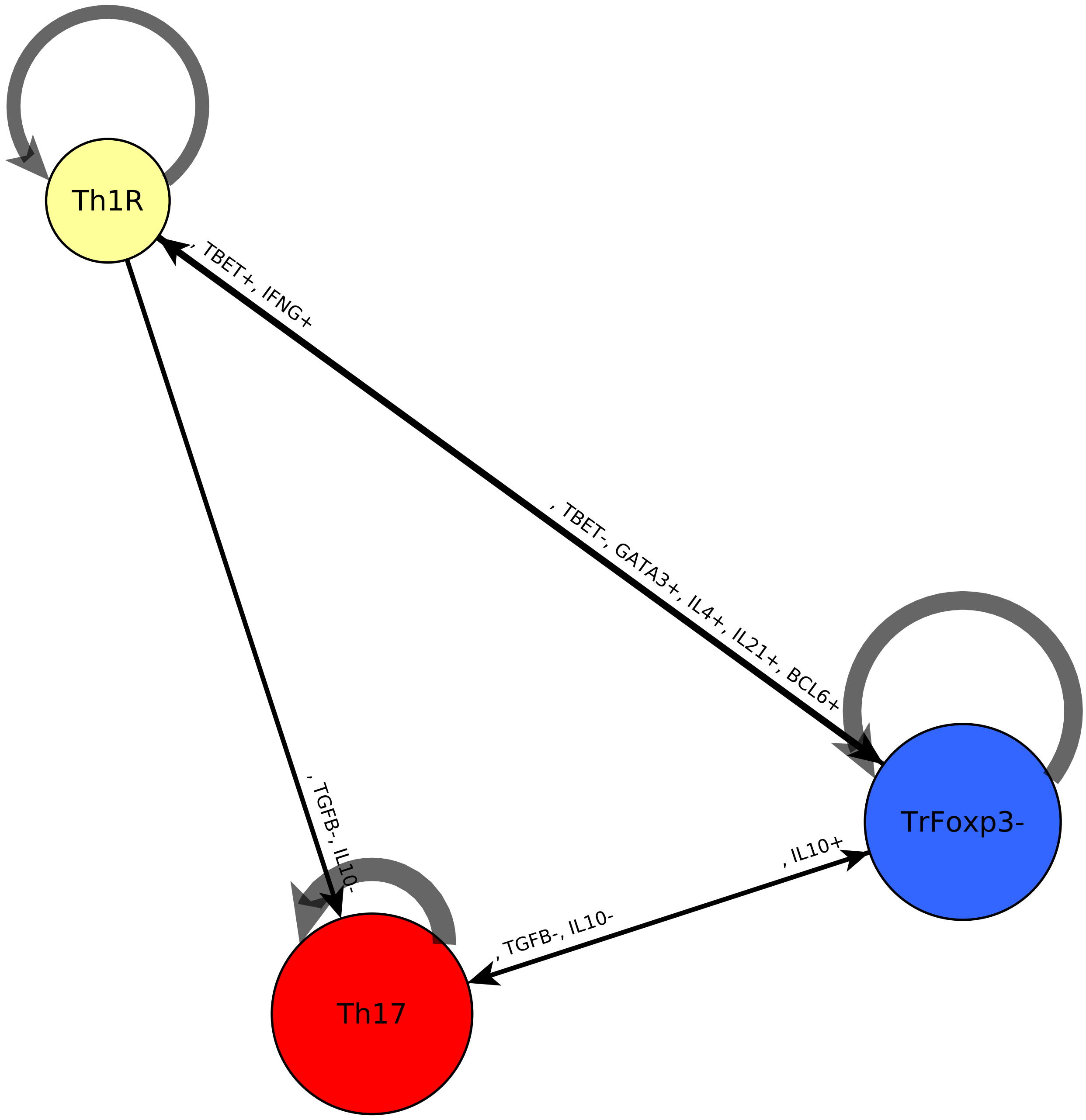

iTreg (TGFβe+ and IL-2e+)

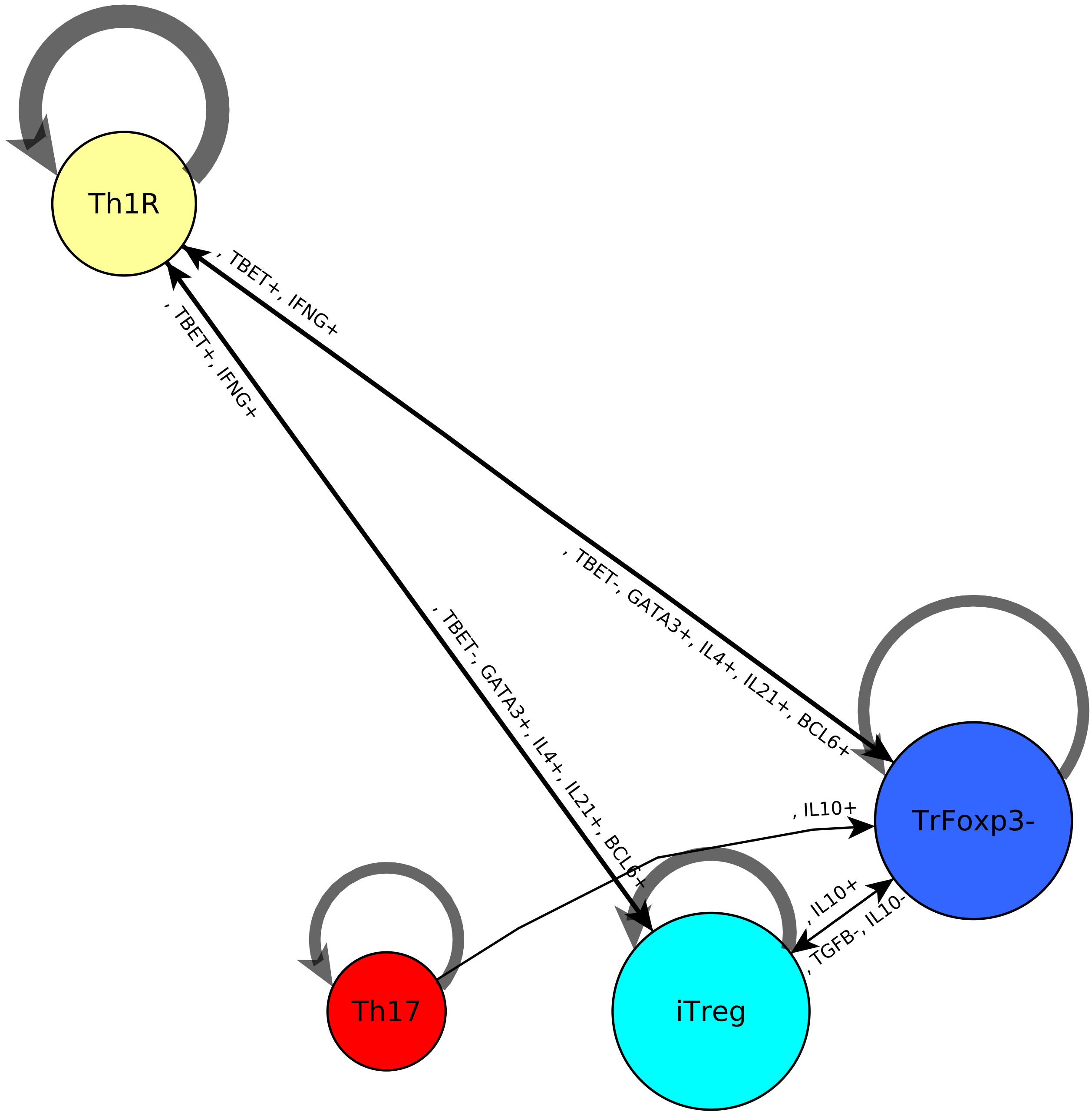

IL-10 (IL-10_e+)

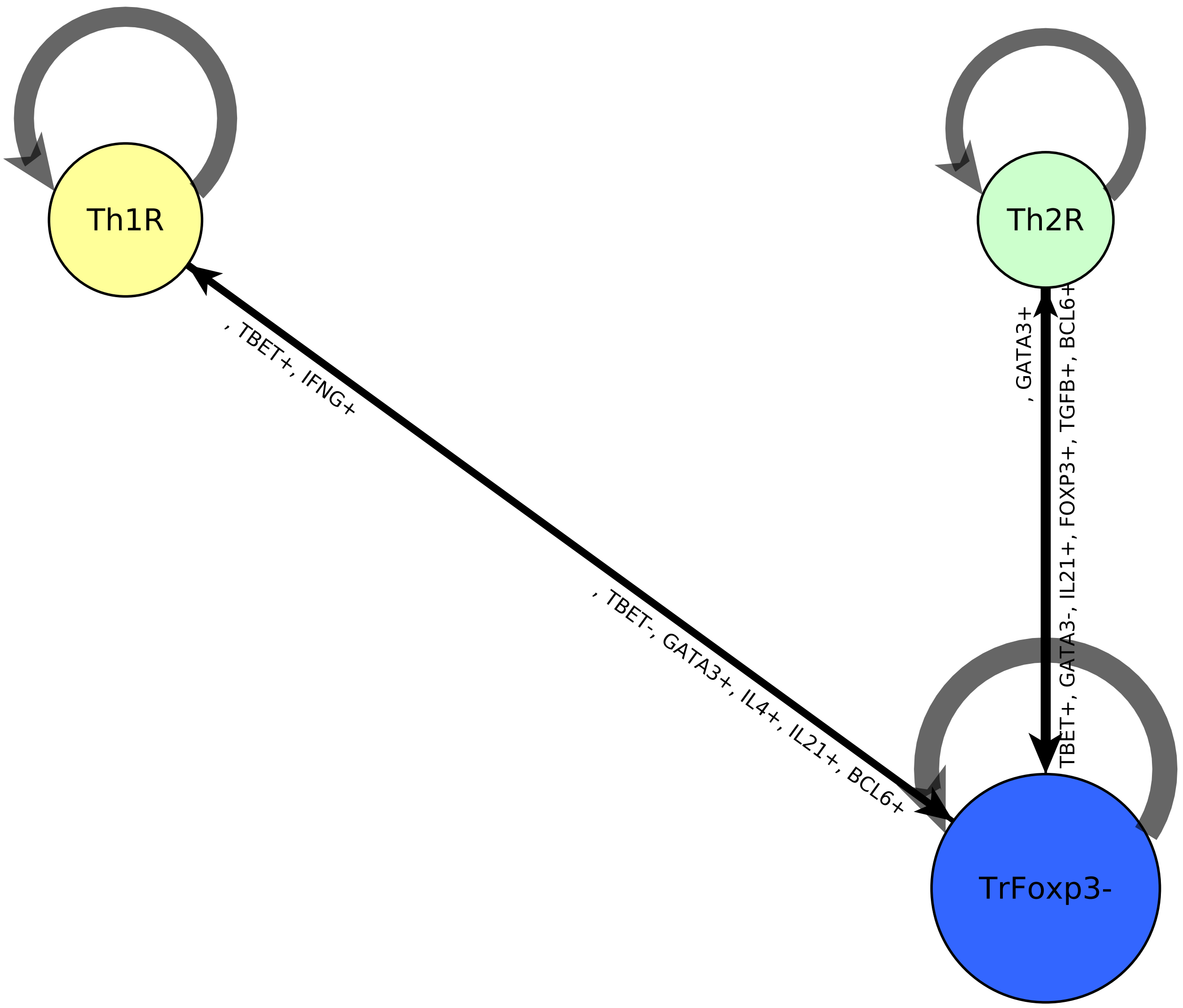

Th1R
Th2R
TrFoxp3-
, TBET+, IFNG+
, TBET-, GATA3+, IL4+, IL21+, BCL6+
, GATA3+
TBET+, GATA3-, IL21+, FOXP3+, TGFB+, BCL6+

Tfh (IL-21_e+)

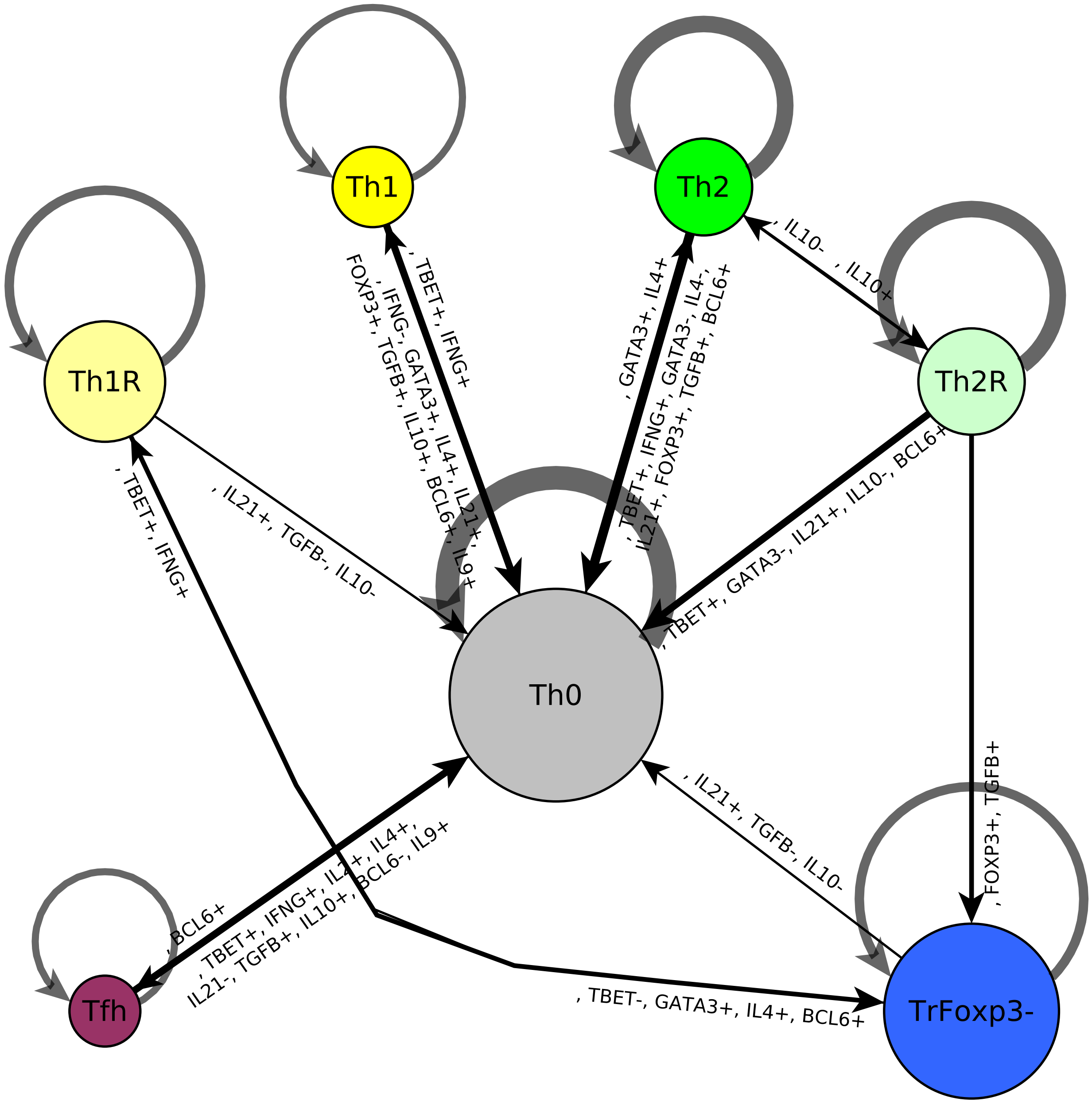
Th1
Th2
Th1R
Th2R
Th0
Tfh
TrFoxp3-
, TBET+, IFNG+
, IFNG-, GATA3+, IL4+, IL21+,
FOXP3+, TGFB+, IL10+, BCL6+, IL9+
, GATA3+, IL4+
, TBET+, IFNG+, GATA3-, IL4-,
IL21+, FOXP3+, TGFB+, BCL6+
, IL10-
, IL10+
, TBET+, GATA3-, IL21+, IL10-, BCL6+
, IL21+, TGFB-, IL10-
, TBET+, IFNG+
, FOXP3+, TGFB+
, IL21+, TGFB-, IL10-
, BCL6+
, TBET+, IFNG+, IL2+, IL4+,
IL21-, TGFB+, IL10+, BCL6-, IL9+
, TBET-, GATA3+, IL4+, BCL6+

IL-9 (IL-4_e+ and TGFβe)

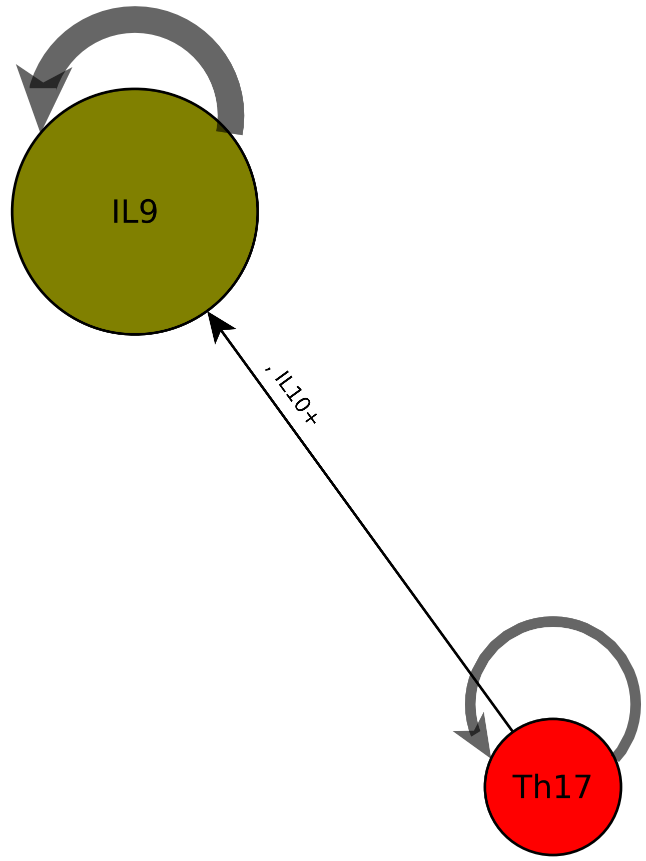

SOCS-

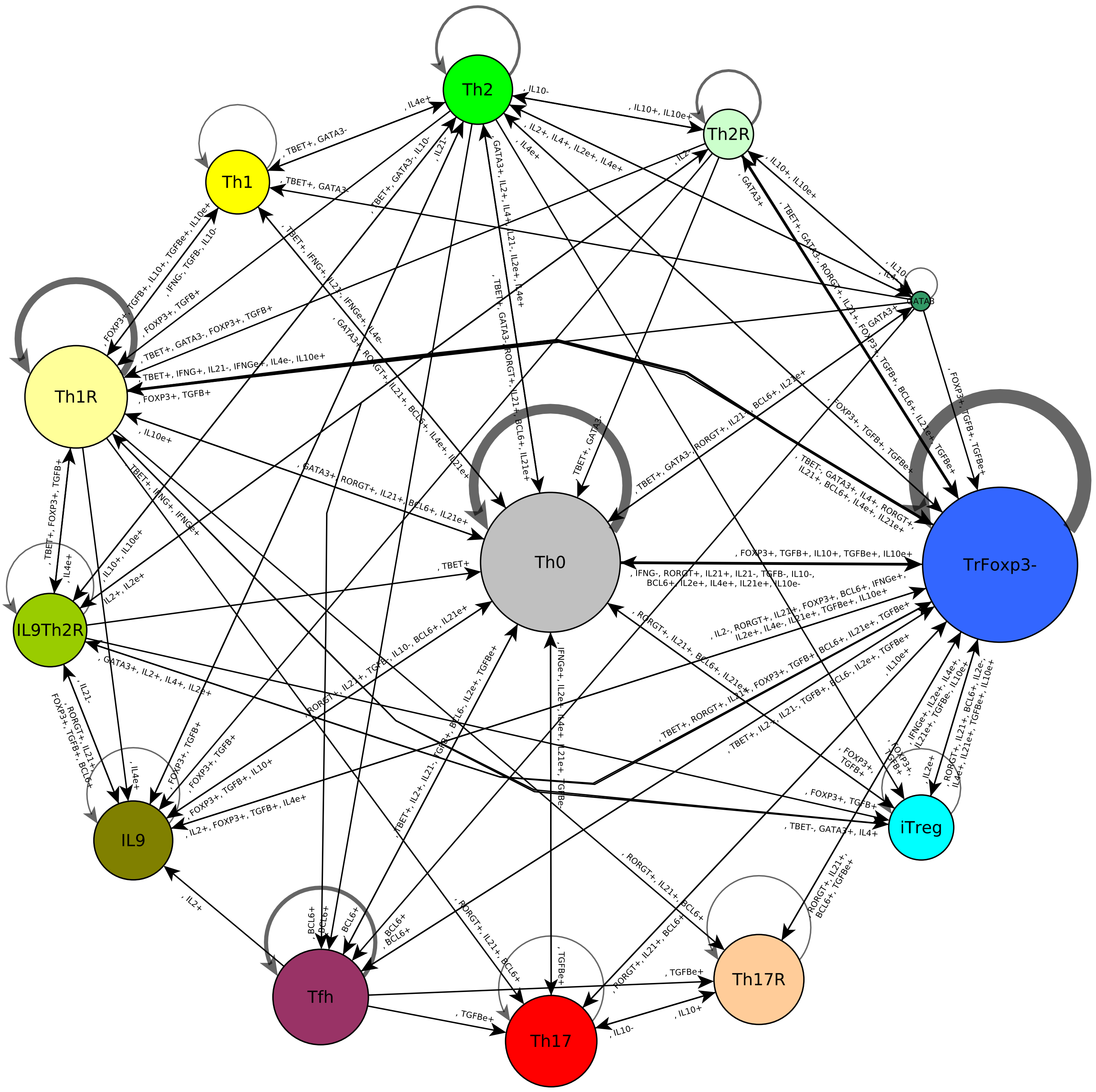
Th2
Th2R
Th1
GATA3
Th1R
Th0
TrFoxp3-
IL9Th2R
iTreg
IL9
Tfh
Th17
Th17R
